# Clinical Outcomes and Medical Costs of Hospitalized Children Requiring Daily Medical Care in Japan

**DOI:** 10.2188/jea.JE20240457

**Published:** 2025-12-05

**Authors:** Osamu Matsumura Momo, Susumu Kunisawa, Kenji Kishimoto, Kiyohide Fushimi, Yuichi Imanaka

**Affiliations:** 1 Department of Healthcare Economics and Quality Management, School of Public Health, Graduate School of Medicine, Kyoto University, Kyoto, Japan; 2 Department of Health Policy and Informatics, Institute of Science Tokyo Graduate School of Medical and Dental Sciences, Tokyo, Japan; 3 Department of Health Security System, Center for Health Security, Graduate School of Medicine, Kyoto University, Kyoto, Japan

**Keywords:** hospital pediatrics, children with medical complexity, technology-dependent children, readmission rate, medical cost

## Abstract

**Background:**

This study aimed to describe the clinical outcomes and medical costs of hospitalized children requiring daily medical care (CRDMC), a patient group for which government-led support has developed rapidly in Japan.

**Methods:**

A retrospective longitudinal study was conducted using a nationwide administrative database. All hospitalizations of children aged under 18 years discharged from April 2014 to March 2021 were included. Clinical outcomes and medical costs were compared between CRDMC and non-CRDMC hospitalizations. The estimated increase in the proportion of CRDMC medical costs among all pediatric hospitalizations during the study period was also calculated.

**Results:**

Among the 1,531,456 hospitalizations included, 91,413 were identified as CRDMC. CRDMC accounted for 3.7% of the annual unique inpatients. The 30-day readmission rate among CRDMC was 27.5%, and the rate among those receiving multiple types of medical care was higher at 33.7%. The inpatient medical cost of CRDMC accounted for 20.3% of pediatric inpatient medical costs, with an estimated rise of 1.2881 (95% confidence interval, 1.2110–1.3702) during the study period. In the breakdown of the medical costs, the proportion of injection drug fees increased most rapidly.

**Conclusion:**

The high 30-day readmission rate in CRDMC was distinctive among the clinical outcomes. The proportion of medical costs for CRDMC in pediatric inpatients was high, although CRDMC accounted for only a small proportion of annual unique inpatients. Further support for CRDMC must be based on the unique characteristics of this population.

## INTRODUCTION

Nowadays, pediatric research focuses on children with medical complexity (CMC), defined as those with severe and chronic clinical conditions, substantial family-identified healthcare service needs, major functional limitations, and high health resources utilization.^1^^–^^3^ Recently, a subgroup of CMC^4^ who require medical care in daily life, referred to as children requiring daily medical care (CRDMC, “*Iryou-teki-Care-Ji*” in Japanese), came into the spotlight in Japan due to their high demands for social support. To meet the demand, the “Act on Supporting CRDMC and Their Families”^5^ was passed in 2021. In the Act, the term CRDMC was formally defined as “children (those under 18 years old or in high school education) who require medical care on a daily basis, such as respiratory management with ventilator or sputum suction, in order to live their daily or social lives.” This definition is in a sense similar to a different subgroup of CMC called technology-dependent children, which was defined by the United States Office of Technology Assessment as “one who needs both a medical device to compensate for the loss of a vital body function and substantial and ongoing nursing care to avert death or further disability”.^6^^–^^8^ However, these definitions differ in that CRDMC focuses on the need for medical care on a daily basis, regardless of the use of a medical device. Furthermore, the purpose of medical care for CRDMC is not limited only to biological survival, but to maintaining daily and social lives as well. Therefore, CRDMC represent a unique patient group with distinct needs that should be identified by a dedicated algorithm.

Understanding the characteristics and patterns of healthcare utilization by CRDMC is essential for planning healthcare policies and assessing their impact. Previous studies targeting CRDMC^9^^–^^15^ have revealed several important features, including a prevalence of 0.99 to 1.88 per 1,000 Japanese children and an increasing trend in recent years.^9^^,^^14^ However, studies on CRDMC in hospital care are lacking. Prior studies revealed that CMC, particularly those with multiple chronic conditions, have a higher risk of re-admission,^16^^–^^18^ death,^19^ a longer length of stay,^20^ and a large and increasing proportion of medical costs,^21^^–^^26^ especially for in-hospital care.^2^^,^^26^^–^^28^ Yet, whether or not these are also true for the CRDMC population is unknown. Therefore, this study aimed to describe the clinical outcomes and medical costs of hospitalized CRDMC and evaluate the financial impact of this population.

## METHODS

### Study design

We conducted a retrospective longitudinal study using the Diagnosis Procedure Combination (DPC) administrative database. The DPC Research Group, funded by Japan’s Ministry of Health, Labour and Welfare, collected data based on the Japanese prospective payment system applied to acute care hospitals called the DPC/per-diem payment system (PDPS).^29^ For approximately 7 million patients per year from more than 1,000 hospitals in Japan, the database contains hospital data, inpatient and outpatient procedure claims data, equipment and drugs, and inpatient summaries upon discharge.^30^^,^^31^

### Participants

Data from hospitals that provided both inpatient and outpatient data every month between April 1, 2013, and March 31, 2022, was extracted from the DPC database. All extracted data were used for identifying CRDMC. All hospitalizations of children younger than 18 years of age at admission who were discharged between April 1, 2014 (the first day of fiscal year [FY] 2014 in Japan) and March 31, 2021 (the last day of FY 2020 in Japan) were included in the study. The discharge date of each hospitalization was used as the index date. All hospitalizations, including those of the same patients, were analyzed as independent hospitalizations. Hospitalizations with missing claims data were excluded from the study.

### Identifying children requiring daily medical care

In accordance with the “Act on Supporting CRDMC and Their Families”,^5^ CRDMC were defined as children under 18 years old who were receiving medical care on a daily basis, regardless of whether at home (home-cared CRDMC), in a medical facility (in-hospital CRDMC), in a nursing home, or other facilities. We included the following categories of medical care^9^^,^^14^: respiratory care (including invasive mechanical ventilation, non-invasive mechanical ventilation, continuous positive airway pressure, tracheostomy, oxygen therapy, and sputum suction), tube feeding (including nasal tube feeding, oral tube feeding, care of gastrostomy or enterostomy, and percutaneous trans-esophageal gastro-tubing), central parenteral nutrition, urinary care (including urethral catheterization, care of nephrostomy, urostomy or vesicostomy), defecation care (including intestinal cleansing and care of colostomy), dialysis (including blood dialysis and peritoneal dialysis), congenital intractable skin disease care (including care for epidermolysis bullosa and bullous congenital ichthyosiform erythroderma). We identified CRDMC patients who met one or more of the following requirements:

1. patients with home medical care who are supervised or provided with their medical equipment by the physician at the hospital of hospitalization within 365 days before or after discharge;
2. patients who required medical care for more than four consecutive weeks during the hospitalization, with no special/specified hospital fees or additional fees for the maternal-fetal intensive care unit, neonatal intensive care unit, growing care unit, intensive care unit, high care unit, or emergency care ward within that period;
3. patients who required certain medical care upon discharge to their home or a nursing home;
4. patients who had surgery during hospitalization to install or uninstall a medical device used for medical care;
5. patients who were charged an extra fee for scoring ten or more points on the Severe Motor and Intellectual Disabilities-Medical Care Dependent Group Scoring System,^32^ which suggested that the patient was receiving at least one of the listed types of medical care.

The first requirement was set to identify home-cared CRDMC, the second requirement was set to identify in-hospital CRDMC, and the third to fifth requirements were set to supplement the first and second requirements. Congenital intractable skin disease care was included only in the first requirement. The details of the requirements are provided in eMaterial 1 and the medical codes used for identification are listed on eTable 1.

### Study measures

Outcomes included dispositions (in-hospital mortality and site of discharge), 30-day readmission rate, 30-day non-elective readmission (readmission not intended) rate, medical costs, and length of stay. Medical costs included medical cost per hospitalization, medical cost per day, the breakdown of medical costs, the proportion of inpatient CRDMC medical costs, the breakdown of inpatient CRDMC medical costs, and inpatient CRDMC health insurance payments in all pediatric hospitalizations each month of the study period. We calculated the medical cost per day and its breakdown by dividing the medical cost per hospitalization by hospital days and rounding up to the nearest whole number for each hospitalization. We calculated the monthly proportion by dividing the sum of the medical costs of CRDMC discharged each month by the sum of the medical costs of all pediatric inpatients discharged in the same month. We broke down the medical costs into the following categories: laboratory and histopathological examinations, imaging tests, oral and topical drugs, injection drugs, medical procedures, surgeries, first visit fees, medical supervision fees, home medical care-related fees, basic hospital fees, special or specified hospital fees, and other.

### Medical costs

We used fee-for-service equivalent costs as medical costs. Fee-for-service equivalent medical costs are the sum of all costs that would be billed during hospitalization if the payment were based on a fee-for-service system. Fee-for-service equivalent costs could have discrepancies with health insurance payments since a large portion of health insurance payments are calculated by the product of per-diem fees for each DPC code, hospital days, and coefficients specific to medical institutions.^29^

### Statistical analysis

To summarize the study population, we described the distribution of age, sex, and weight on admission as well as admission status (whether the admission was directly after birth, on emergency, scheduled, or other), diagnoses of common acute diseases (whether the applicable ICD-10 codes^33^^,^^34^ were in the main diagnosis, admission-precipitating diagnosis, or most resource-consuming diagnosis), the type of medical care received, and diagnoses of specific pediatric chronic diseases.^35^ We used the Japanese list of specific pediatric chronic diseases as of November 1, 2021^36^ to identify the diagnoses of these diseases by matching the standard disease name(s) (or International Classification of Diseases-10^th^ revision codes for diseases without standard disease names) to all diagnoses recorded during each hospitalization, excluding suspected diagnoses.

We calculated descriptive statistics to summarize clinical outcomes (in-hospital mortality, site of discharge, 30-day readmission rate, and 30-day non-elective readmission rate) and medical costs (medical cost per hospitalization, medical cost per day, and length of stay) of CRDMC and their subpopulations to identify those at high risk of adverse outcomes and with high demands for medical resources. The subpopulations of CRDMC were classified as CRDMC with a single category of medical care, CRDMC with multiple categories of medical care (eg, respiratory care, tube feeding), home-cared CRDMC (identified in the first requirement), and in-hospital CRDMC (identified in the second requirement). We also summarized the breakdown of medical costs per day to describe the use of each component of medical resources. We compared outcome risks and medical costs between CRDMC and non-CRDMC using generalized linear models (GLM) adjusted by age, sex, admission status (as categorized above), and disease groups of specific pediatric chronic diseases to estimate the overall risk of outcomes for CRDMC upon admission. We used multivariate logistic regression to calculate the odds ratio between CRDMC and non-CRDMC on dichotomous and categorical variables and GLM with a quasi-Poisson distribution and log-link function^37^^,^^38^ to calculate the ratio between them on integer variables.

We also used GLM with a quasi-Poisson distribution and log-link function to estimate the increasing proportion of (1) inpatient CRDMC medical costs, (2) the breakdown of inpatient CRDMC medical costs, and (3) inpatient CRDMC health insurance payments in all pediatric hospitalizations from April 2014 to March 2021.

We performed subgroup analyses on the clinical outcomes and medical costs of CRDMC for each type of medical care and medical device. As a sensitivity analysis, we calculated the rate of increase in medical fees using hospitalizations discharged by FY 2019 to determine whether the coronavirus disease 2019 pandemic affected the results.

All missing values were excluded from the analysis. Columns fewer than ten hospitalizations were concealed to protect personal information. Analyses were performed using R Statistical Software version 4.2.1 (R Foundation for Statistical Computing, Vienna, Austria).

### Ethics statement

This study was approved by the Ethics Committee, Graduate School of Medicine, Kyoto University (approval number: R0135).

## RESULTS

**Figure 1.**
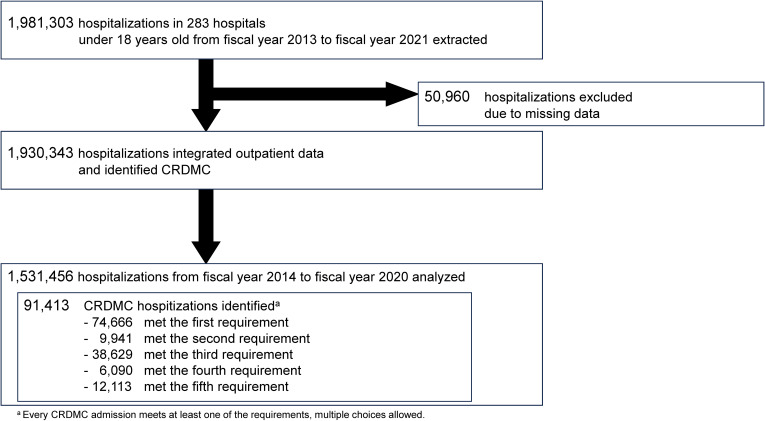
Flow chart of study participants. CRDMC, children requiring daily medical care.

Of the 1,531,456 hospitalizations across 283 hospitals included in this study, 91,413 were identified as CRDMC hospitalizations. The process of selection and exclusion is detailed in Figure 1. Hospital characteristics are shown in eTable 2. Among hospitalized CRDMC, respiratory care was the most common medical care, applied in 56,164 (61.4%) patients, followed by tube feeding in 51,386 (56.2%) patients (Table 1 and eTable 3). Proportions of CRDMC in total hospitalizations, total annual unique patients, and total hospital days were 6.0%, 3.7%, and 17.8%, respectively, with increases observed during the study period (Table 2).

**Table 1.** Characteristics of non-CRDMC, CRDMC, and CRDMC subpopulation hospitalizations

Characteristic	Overall *N* = 1,531,456	Non-CRDMC, *n* = 1,440,043	CRDMC, *n* = 91,413	Subpopulations of CRDMC			

				Single or multiple types of care		Place of care	

				CRDMC with a single type of medical care, *n* = 54,344	CRDMC with multiple types of medical care, *n* = 34,569	Home-cared CRDMC, *n* = 74,666	In-hospital CRDMC, *n* = 9,941
Age, median (IQR), years	3 (0–8)	3 (0–8)	3 (0–8)	2 (0–8)	4 (1–9)	3 (1–8)	1 (0–8)
<1 year, number (%)	449,449 (29.3)	425,550 (29.6)	23,899 (26.1)	16,699 (30.7)	6,262 (18.1)	17,439 (23.4)	4,704 (47.3)
1–2 years, number (%)	306,149 (20.0)	285,998 (19.9)	20,151 (22.0)	11,966 (22.0)	7,871 (22.8)	17,986 (24.1)	1,156 (11.6)
3–5 years, number (%)	249,199 (16.3)	233,652 (16.2)	15,547 (17.0)	8,498 (15.6)	6,606 (19.1)	13,292 (17.8)	936 (9.4)
6–12 years, number (%)	311,667 (20.4)	291,860 (20.3)	19,807 (21.7)	10,009 (18.4)	9,199 (26.6)	16,364 (21.9)	1,646 (16.6)
13 years or older, number (%)	214,992 (14.0)	202,983 (14.1)	12,009 (13.1)	7,172 (13.2)	4,631 (13.4)	9,585 (12.8)	1,499 (15.1)
Sex, number (%)							
Male	864,487 (56.4)	815,331 (56.6)	49,156 (53.8)	29,680 (54.6)	18,012 (52.1)	40,217 (53.9)	5,075 (51.1)
Female	666,969 (43.6)	624,712 (43.4)	42,257 (46.2)	24,664 (45.4)	16,557 (47.9)	34,449 (46.1)	4,866 (48.9)
Weight, median (IQR), kg^a^	13.0 (7.2–24.8)	13.0 (7.3–25.4)	10.9 (6.8–18.2)	10.3 (6.4–18.1)	11.9 (7.6–18.4)	11.0 (7.2–18.1)	8.0 (2.9–19.6)
<3 kg, number (%)	166,920 (11.1)	158,835 (11.2)	8,085 (9.0)	5,325 (10.0)	2,210 (6.6)	4,498 (6.1)	2,556 (26.0)
3–<15 kg, number (%)	342,951 (22.8)	321,457 (22.8)	21,494 (24.0)	10,625 (19.9)	10,209 (30.3)	18,062 (24.6)	1,802 (18.4)
15–<30 kg, number (%)	683,049 (45.5)	631,903 (44.7)	51,146 (57.1)	31,030 (58.0)	18,961 (56.3)	44,054 (59.9)	4,048 (41.2)
30–<50 kg, number (%)	176,874 (11.8)	169,995 (12.0)	6,879 (7.7)	4,803 (9.0)	1,984 (5.9)	5,445 (7.4)	1,045 (10.6)
50 kg or heavier, number (%)	132,793 (8.8)	130,763 (9.3)	2,030 (2.3)	1,722 (3.2)	303 (0.9)	1,446 (2.0)	364 (3.7)
Admission, number (%)^b^							
- Directly after birth	215,098 (14.0)	208,035 (14.4)	7,063 (7.7)	4,735 (8.7)	1,800 (5.2)	3,765 (5.0)	2,272 (22.9)
Born at 32 weeks of gestation or before^c^	14,110 (0.9)	11,707 (0.8)	2,403 (2.6)	1,529 (2.8)	594 (1.7)	1,187 (1.6)	964 (9.7)
- Emergency	748,667 (48.9)	707,980 (49.2)	40,687 (44.5)	21,886 (40.3)	17,685 (51.2)	34,915 (46.8)	4,437 (44.6)
- Scheduled	562,995 (36.8)	519,539 (36.1)	43,456 (47.5)	27,594 (50.8)	15,020 (43.4)	35,854 (48.0)	3,167 (31.9)
- Other	4,694 (0.3)	4,487 (0.3)	207 (0.2)	129 (0.2)	64 (0.2)	132 (0.2)	65 (0.7)
Diagnosis, Number (%)							
- Upper Respiratory Infections	52,393 (3.4)	50,887 (3.5)	1,506 (1.6)	909 (1.7)	548 (1.6)	1,388 (1.9)	17 (0.2)
- Lower Respiratory Infections, influenza, or COVID-19	223,568 (14.6)	210,376 (14.6)	13,192 (14.4)	6,417 (11.8)	6,441 (18.6)	11,831 (15.8)	488 (4.9)
- Gastrointestinal infections	64,084 (4.2)	62,151 (4.3)	1,933 (2.1)	1,177 (2.2)	681 (2.0)	1,780 (2.4)	79 (0.8)
- Urinary tract infections	20,329 (1.3)	18,261 (1.3)	2,068 (2.3)	1,300 (2.4)	738 (2.1)	1,906 (2.6)	52 (0.5)
- Skin, soft tissue infections	14,884 (1.0)	14,637 (1.0)	247 (0.3)	130 (0.2)	114 (0.3)	229 (0.3)	18 (0.2)
- Asthma	57,247 (3.7)	55,265 (3.8)	1,982 (2.2)	1,185 (2.2)	719 (2.1)	1,797 (2.4)	61 (0.6)
- Epilepsies and seizures	67,549 (4.4)	63,199 (4.4)	4,350 (4.8)	2,245 (4.1)	1,940 (5.6)	3,786 (5.1)	296 (3.0)
Type of medical care, number (%)^d^							
- Respiratory care	56,164 (3.7)	—	56,164 (61.4)	25,240 (46.4)	30,924 (89.5)	49,532 (66.3)	6,465 (65.0)
- Tube feeding	51,386 (3.4)	—	51,386 (56.2)	18,765 (34.5)	32,621 (94.4)	42,217 (56.5)	6,945 (69.9)
- Urinary care	8,845 (0.6)	—	8,845 (9.7)	4,019 (7.4)	4,826 (14.0)	7,839 (10.5)	626 (6.3)
- Central parenteral nutrition	6,386 (0.4)	—	6,386 (7.0)	2,836 (5.2)	3,550 (10.3)	4,549 (6.1)	1,931 (19.4)
- Defecation care	3,904 (0.3)	—	3,904 (4.3)	2,275 (4.2)	1,629 (4.7)	2,594 (3.5)	335 (3.4)
- Dialysis	1,787 (0.1)	—	1,787 (2.0)	1,116 (2.1)	671 (1.9)	1,604 (2.1)	393 (4.0)
- Congenital intractable skin disease care	104 (0.0)	—	104 (0.1)	93 (0.2)	11 (0.0)	104 (0.1)	—
- Unknown^e^	2,500 (0.2)	—	2,500 (2.7)	—^f^	—^f^	1,527 (2.0)	—^f^
Diagnosed with any specific pediatric chronic diseases	388,739 (25.4)	329,460 (22.9)	59,279 (64.8)	34,452 (63.4)	23,426 (67.8)	50,910 (68.2)	6,883 (69.2)
- Malignant neoplasm	52,460 (3.4)	46,885 (3.3)	5,575 (6.1)	3,695 (6.8)	1,707 (4.9)	4,070 (5.5)	1,327 (13.3)
- Chronic kidney disease	33,218 (2.2)	29,360 (2.0)	3,858 (4.2)	2,692 (5.0)	1,133 (3.3)	3,306 (4.4)	392 (3.9)
- Chronic respiratory disease	144,926 (9.5)	130,087 (9.0)	14,839 (16.2)	7,447 (13.7)	7,015 (20.3)	13,308 (17.8)	1,364 (13.7)
- Chronic heart disease	68,694 (4.5)	50,587 (3.5)	18,107 (19.8)	12,532 (23.1)	5,339 (15.4)	15,994 (21.4)	2,493 (25.1)
- Endocrine disease	20,458 (1.3)	15,573 (1.1)	4,885 (5.3)	2,359 (4.3)	2,414 (7.0)	4,425 (5.9)	610 (6.1)
- Connective tissue disease	7,219 (0.5)	6,941 (0.5)	278 (0.3)	206 (0.4)	70 (0.2)	234 (0.3)	54 (0.5)
- Diabetes mellitus	6,541 (0.4)	6,153 (0.4)	388 (0.4)	234 (0.4)	151 (0.4)	326 (0.4)	62 (0.6)
- Inborn error of metabolism	10,570 (0.7)	7,032 (0.5)	3,538 (3.9)	1,548 (2.8)	1,834 (5.3)	3,323 (4.5)	235 (2.4)
- Hematologic disease	10,323 (0.7)	9,591 (0.7)	732 (0.8)	503 (0.9)	223 (0.6)	547 (0.7)	223 (2.2)
- Immune disease	11,554 (0.8)	9,661 (0.7)	1,893 (2.1)	1,396 (2.6)	461 (1.3)	1,399 (1.9)	435 (4.4)
- Neuromuscular disease	30,523 (2.0)	20,168 (1.4)	10,355 (11.3)	4,343 (8.0)	5,648 (16.3)	8,837 (11.8)	856 (8.6)
- Chronic digestive disease	17,922 (1.2)	13,096 (0.9)	4,826 (5.3)	2,817 (5.2)	1,898 (5.5)	4,138 (5.5)	476 (4.8)
- Syndrome involving chromosomal or genetic alterations	16,376 (1.1)	9,342 (0.6)	7,034 (7.7)	3,303 (6.1)	3,645 (10.5)	6,263 (8.4)	861 (8.7)
- Skin disease	1,675 (0.1)	1,452 (0.1)	223 (0.2)	155 (0.3)	66 (0.2)	164 (0.2)	14 (0.1)
- Skeletal dysplasia	3,373 (0.2)	2,941 (0.2)	432 (0.5)	206 (0.4)	221 (0.6)	346 (0.5)	63 (0.6)
- Vascular disease	2,619 (0.2)	2,312 (0.2)	307 (0.3)	205 (0.4)	102 (0.3)	271 (0.4)	26 (0.3)

COVID-19, coronavirus disease 2019; CRDMC, children requiring daily medical care; IQR, interquartile range.

^a^Missing data for weight: 27,090 in the non-CRDMC hospitalizations, 1,779 in the CRDMC hospitalizations, 839 in CRDMC receiving a single type of medical care, 902 in CRDMC receiving multiple types of medical care, 1,161 in home-cared CRDMC, and 126 in in-hospital CRDMC.

^b^Missing data for admission: 2 in the non-CRDMC hospitalizations, and 0 in the CRDMC hospitalizations.

^c^Missing data for “Born at 32 weeks of gestation or before”: 552 in the non-CRDMC hospitalizations, 51 in the CRDMC hospitalizations, 23 in CRDMC receiving a single type of medical care, 25 in CRDMC receiving multiple types of medical care, 29 in home-cared CRDMC, and 25 in in-hospital CRDMC.

^d^Multiple choices allowed, except for “Unidentifiable.”

^e^Identified that the patient requires medical care, although the details of the care were not specified.

^f^Columns with under ten hospitalizations were concealed to protect personal information.

**Table 2.** The proportion of CRDMC in total hospitalizations, total annual unique patients, and total hospital days, by discharge fiscal year

	Overall	Fiscal year							Estimated increase rate per year

		2014	2015	2016	2017	2018	2019	2020	
Hospitalizations									
-All pediatric patients	1,531,456	222,294	230,213	228,352	229,996	230,515	225,668	164,418	
-CRDMC	91,413	11,713	12,807	13,284	13,838	13,942	13,970	11,859	
-Proportion of CRDMC	6.0%	5.3%	5.6%	5.8%	6.0%	6.0%	6.2%	7.2%	1.0423 (1.0270–1.0578)
Annual unique patients									
-All pediatric patients	1,250,702^a^	184,167	190,672	187,563	187,055	186,421	182,317	132,507	
-CRDMC	45,816^a^	6,006	6,624	6,719	6,764	6,796	6,862	6,045	
-Proportion of CRDMC	3.7%	3.3%	3.5%	3.6%	3.6%	3.6%	3.8%	4.6%	1.0415 (1.0198–1.0638)
Hospital days									
-All pediatric patients	14,388,327	2,173,064	2,189,654	2,130,484	2,099,473	2,075,207	2,041,895	1,678,550	
-CRDMC	2,563,518	359,698	372,387	364,390	364,812	371,688	383,525	347,018	
-Proportion of CRDMC	17.8%	16.6%	17.0%	17.1%	17.4%	17.9%	18.8%	20.7%	1.0326 (1.0205–1.0450)

CRDMC, children requiring daily medical care.

^a^Sum of the annual unique patients from fiscal year 2014 to fiscal year 2020.

In CRDMC hospitalizations, the in-hospital mortality was 1.9%, the 30-day readmission rate was 27.5%, and the 30-day non-elective readmission rate was 12.4%. Adjusted odds ratios of in-hospital mortality, 30-day readmission rate, and 30-day non-elective readmission rate of CRDMC compared to non-CRDMC were 4.810 (95% confidence interval [CI], 4.490–5.152), 3.746 (95% CI, 3.675–3.817) and 4.117 (95% CI, 4.012–4.224), respectively. The mean medical cost per hospitalization was higher among CRDMC hospitalizations than in non-CRDMC hospitalizations, resulting from both the higher mean medical cost per day and longer mean length of stay. CRDMC with multiple types of medical care had a higher risk of adverse outcomes and medical costs than CRDMC with a single type of medical care (Table 3). Among the types of medical care, respiratory care had the greatest impact on adverse outcomes and medical costs, while hospitalizations with central parenteral nutrition carried a high risk of adverse outcomes. Among medical devices, tracheostomy had both the highest impact on and highest risk of adverse outcomes and medical costs (eTable 4).

**Table 3.** Clinical outcomes and medical costs of non-CRDMC, CRDMC, and CRDMC subpopulations

Characteristic	Overall *N* = 1,531,456	Non-CRDMC, *n* = 1,440,043	CRDMC, *n* = 91,413	Adjusted odds ratio^c^ (95% CI)	Subpopulations of CRDMC			

					Single or multiple types of care		Place of care	

					CRDMC with a single type of medical care, *n* = 54,344	CRDMC with multiple types of medical care, *n* = 34,569	Home-cared CRDMC, *n* = 74,666	In-hospital CRDMC, *n* = 9,941
Disposition, number (%)^a^								
- Death (in-hospital mortality)	5,329 (0.3)	3,600 (0.2)	1,729 (1.9)	4.810 (4.490–5.152)	731 (1.3)	932 (2.7)	692 (0.9)	992 (10.0)
- Discharge to nursing home	2,751 (0.2)	2,296 (0.2)	455 (0.5)	2.937 (2.618–3.288)	240 (0.4)	203 (0.6)	276 (0.4)	76 (0.8)
- Transferred to another hospital	29,786 (1.9)	25,689 (1.8)	4,097 (4.5)	1.923 (1.850–1.998)	2,208 (4.1)	1,641 (4.7)	2,221 (3.0)	1,638 (16.5)
- Discharge to home	1,489,844 (97.3)	1,404,797 (97.6)	85,047 (93.0)	0.439 (0.426–0.454)	51,118 (94.1)	31,759 (91.9)	71,406 (95.6)	7,215 (72.6)
- Other/ unidentified	3,739 (0.2)	3,656 (0.3)	83 (0.1)	0.403 (0.320–0.500)	47 (0.1)	33 (0.1)	69 (0.1)	19 (0.2)
30-day readmissions, number (%)	124,789 (8.1)	99,694 (6.9)	25,095 (27.5)	3.746 (3.675–3.817)	12,887 (23.7)	11,648 (33.7)	21,455 (28.7)	2,189 (22.0)
30-day non-elective readmissions, number (%)^b^	52,208 (3.4)	40,919 (2.8)	11,289 (12.4)	4.117 (4.012–4.224)	5,208 (9.6)	5,869 (17.0)	10,595 (14.2)	1,072 (10.8)

				Adjusted ratio^c^ (95% CI)				

Medical cost per hospitalization, mean (SD), JPY	654,323 (1,906,129)	554,908 (1,329,956)	2,220,424 (5,513,415)	2.930 (2.905–2.955)	1,964,482 (4,458,898)	2,615,751 (6,865,430)	1,664,722 (4,077,037)	10,767,048 (12,094,924)
Medical cost per day, mean (SD), JPY	72,841 (147,415)	70,337 (116,537)	112,291 (385,316)	1.155 (1.147–1.163)	110,312 (338,075)	113,815 (442,002)	113,139 (384,890)	73,549 (60,464)
Length of stay, mean (SD), day	8.4 (23.5)	7.2 (15.9)	27.0 (70.3)	3.138 (3.111–3.165)	21.9 (47.8)	35.3 (96.2)	20.0 (50.2)	148.7 (156.5)

CI, confidence interval; CRDMC, children requiring daily medical care; JPY, Japanese yen; SD, standard deviation.

^a^Missing data for disposition: 5 in the non-CRDMC hospitalizations, 2 in the CRDMC hospitalizations, 0 in CRDMC receiving a single type of medical care, 1 in CRDMC receiving multiple types of medical care, 2 in home-cared CRDMC, and 1 in in-hospital CRDMC.

^b^Missing data for 30-day non-elective readmissions: 150 in the CRDMC hospitalizations, 24 in the non-CRDMC hospitalizations, 11 in CRDMC receiving a single type of medical care, 13 in CRDMC receiving multiple types of medical care, 23 in home-cared CRDMC, and 3 in in-hospital CRDMC.

^c^Adjusted by age, sex, admission status (admission directly after birth, on emergency, scheduled, or other), and disease groups of specific pediatric chronic diseases.

**Figure 2.**
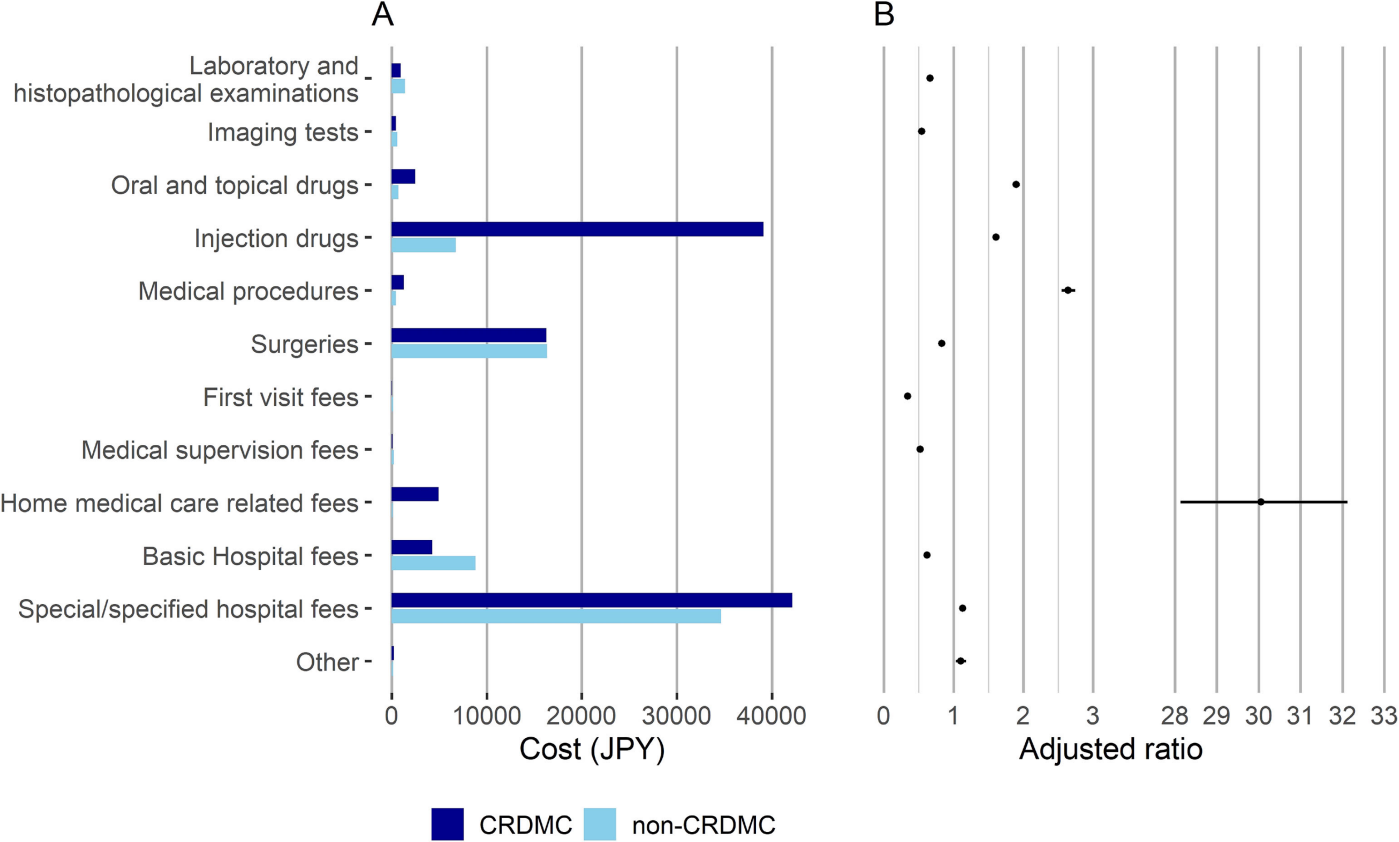
Breakdown of inpatient medical costs per day in CRDMC and non-CRDMC hospitalizations. (**A**) Breakdown of mean inpatient medical costs per day. (**B**) Ratios adjusted for age, sex, admission status, and disease groups of specific pediatric chronic diseases with 95% CI of inpatient medical costs per day in CRDMC to non-CRDMC hospitalizations. Labels are common for panels A and B. CI, confidence interval; CRDMC, children requiring daily medical care.

The breakdown of the mean inpatient medical costs per day was noticeably higher in CRDMC than non-CRDMC hospitalizations for oral and topical drug fees, injection drug fees, medical procedure fees, and home medical care-related fees, with an adjusted ratio of 1.5 or higher (Figure 2 and eTable 5). The breakdown of the mean inpatient medical costs per hospitalization for CRDMC and non-CRDMC hospitalizations is also shown in eTable 6.

**Figure 3.**
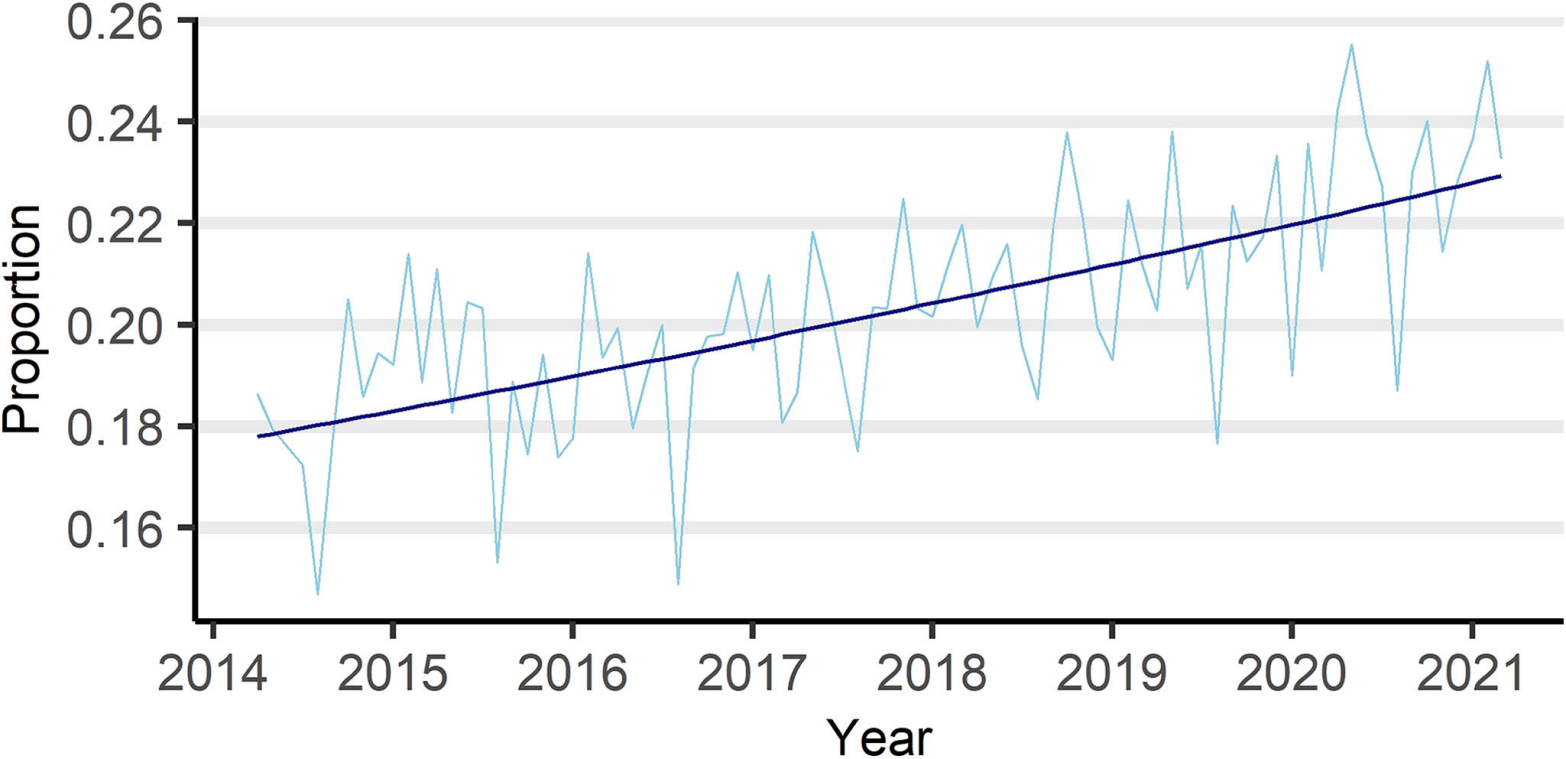
Proportion and estimated increase in the proportion of inpatient CRDMC medical costs among all pediatric hospitalizations, by discharge month. CRDMC, children requiring daily medical care.

Total pediatric inpatient medical costs were 1,002,066,909,679 Japanese yen (JPY) in the 7 years of the study period, whereas CRDMC inpatient medical costs were 202,975,642,099 JPY in the same period, accounting for 20.3% of pediatric inpatient medical costs. The estimated proportion of CRDMC inpatient medical costs in pediatric inpatient medical costs increased from 17.8% (95% CI, 17.2–18.5%) to 22.9% (95% CI, 20.8–25.3%) during the study period, with an estimated rise of 1.2881 (95% CI, 1.2110–1.3702) (Figure 3 and Table 4). The proportional increase was noticeably high in injection drug fees, with an estimated rise of 1.6373 (95% CI, 1.4517–1.8475) during the period. The proportion of CRDMC home medical care-related fees increased sharply in FY 2016, while other components showed a steady trend throughout the period. (eFigure 1 and Table 4). The estimated proportion of health insurance payments increased from 18.2% (95% CI, 17.6–19.0%) to 21.9% (95% CI, 19.7–24.2%), and the estimated rise over the study period was 1.1986 (95% CI, 1.1241–1.2781) (Table 4). The rates of increase estimated from hospitalizations discharged by FY 2019 showed the same trend among the components of medical costs and health insurance payments, although these rates were generally lower than those observed in models including FY 2020 data (eTable 7 and eFigure 2).

**Table 4.** Estimated proportion of inpatient medical costs, the breakdown of medical costs, and health insurance payments of CRDMC hospitalizations among overall pediatric inpatients, by discharge month

	Estimated proportion on April 2014 (95% CI), %	Estimated proportion on March 2021 (95% CI), %	Estimated increase rate per 12 months^a^ (95% CI)	Estimated increase rate over the study period^a^ (95% CI)
Inpatient medical costs	17.8 (17.2–18.5)	22.9 (20.8–25.3)	1.0373 (1.0281–1.0466)	1.2881 (1.2110–1.3702)
- Laboratory and histopathological examinations	17.2 (16.1–18.3)	21.3 (18.0–25.1)	1.0314 (1.0158–1.0471)	1.2380 (1.1145–1.3753)
- Imaging tests	16.1 (15.4–17.0)	20.9 (18.2–23.9)	1.0379 (1.0253–1.0507)	1.2936 (1.1883–1.4082)
- Oral and topical drugs	28.6 (27.2–30.1)	37.3 (32.6–42.7)	1.0391 (1.0265–1.0518)	1.3034 (1.1979–1.4183)
- Injection drugs	24.4 (22.5–26.3)	39.9 (32.7–48.6)	1.0739 (1.0554–1.0928)	1.6373 (1.4517–1.8475)
- Medical procedures	41.6 (39.9–43.2)	48.7 (43.8–54.2)	1.0233 (1.0135–1.0332)	1.1725 (1.0970–1.2533)
- Surgeries	17.5 (16.1–19.0)	19.5 (15.6–24.4)	1.0162 (0.996–1.0369)	1.1177 (0.9724–1.2849)
- First visit fees	3.3 (3.1–3.5)	4.3 (3.7–4.9)	1.0387 (1.0254–1.0521)	1.3003 (1.1896–1.4213)
- Medical supervision fees	10.5 (9.7–11.3)	9.5 (7.7–11.8)	0.9866 (0.967–1.0066)	0.9110 (0.7927–1.0469)
- Home medical care related fees	60.6 (56.5–65.0)	86.7 (72.6–103.4)	1.0531 (1.037–1.0695)	1.4304 (1.2858–1.5918)
- Basic Hospital fees	13.0 (12.1–14.0)	16.2 (13.2–19.9)	1.0322 (1.013–1.0517)	1.2447 (1.0931–1.4172)
- Special/specified hospital fees	17.3 (16.8–17.8)	20.8 (19.3–22.4)	1.0269 (1.0198–1.0341)	1.2016 (1.1449–1.2611)
- Other	20.1 (18.3–22.0)	25.9 (20.2–33.0)	1.0374 (1.0145–1.0608)	1.2889 (1.1044–1.5042)
Health insurance payments	18.2 (17.6–19.0)	21.9 (19.7–24.2)	1.0265 (1.0171–1.0361)	1.1986 (1.1241–1.2781)

CI, confidence interval; CRDMC, children requiring daily medical care; JPY, Japanese yen.

^a^Not adjusted for covariates.

## DISCUSSION

In this retrospective longitudinal study using nationwide administrative inpatient data, we found that CRDMC are at high risk of adverse outcomes and high medical costs. The proportion of CRDMC inpatient medical costs in overall pediatric hospitalizations increased during the study period, with fees for injection drugs showing the highest rate of increase.

CRDMC, especially those with multiple types of medical care, had particularly higher readmission rates compared with those reported previously for CMC. The in-hospital mortality rates in CRDMC with a single type of medical care and CRDMC with multiple types of medical care were 1.3% and 2.7%, respectively, which were similar or slightly lower than the in-hospital mortality rates of 1.3% (one complex chronic condition) and 3.6% (multiple complex chronic conditions) reported by a previous CMC study.^21^ In contrast, readmission rates accounted for more than those reported in CMC. In the current study, 30-day readmissions in CRDMC with single type of medical care and CRDMC with multiple types of medical care were 23.7% and 33.7%, respectively, and 30-day non-elective readmissions in these groups were 9.6% and 17.0%, respectively. These readmission rates were higher than those in prior CMC studies.^18^^,^^39^ Several interventions, such as outpatient visits within 4 to 29 days after discharge^40^ and medical home-like transitional care models,^41^ have proven effective in reducing the readmission rates of CMC; further research on whether these interventions are also effective for CRDMC is desirable.

The adjusted medical cost ratio per hospitalization was nearly 3-fold for CRDMC than non-CRDMC hospitalizations due to longer stays and higher medical costs per day. Consequently, CRDMC accounted for 20.3% of inpatient medical costs, in contrast to only 3.7% in annual unique patients. The estimated proportion of inpatient medical costs of CRDMC in total pediatric inpatient medical costs increased by 1.2881 over the 7 years of the study period, reflecting the rapid growth in the CRDMC population in recent years.^9^^,^^14^ In order to reduce cost growth in the face of population growth, optimizing CRDMC hospital care is essential.

In the breakdown of medical costs, medication-related costs were particularly high and increasing. The adjusted ratios of mean daily medical fees for oral and topical drugs and injection drugs for CRDMC were 1.898-fold and 1.609-fold higher, respectively, than those of non-CRDMC, and the proportion of injection drug fees for CRDMC in the pediatric population increased disproportionately relative to the increase in patient numbers. Issues related to high resource use for medications, such as polypharmacy^42^ and chronic use of multiple medications,^43^ accompanied with medication order errors,^44^ adverse drug events,^45^ and potential drug-drug interactions,^46^ have been noted in CMC. In CMC with severe neurological impairment, it has also been indicated that medication regimens do not necessarily meet their needs.^47^ If these issues also exist in CRDMC, optimizing medications could reduce both medication-related costs and other medical expenses resulting from adverse drug events. Further studies are necessary to investigate whether there is room for improvement in CRDMC medication management.

The proportion of home medical care-related fees also showed a disproportionate increase relative to the increase in patient numbers. However, unlike other components of medical costs, this increase was primarily driven by a sharp increase in 2016, which may have resulted from the biennial revision of medical service fees. From that year, home medical care-related fees expanded for CRDMC, such as the establishment of a post-discharge home visit fee.^48^ Future research is warranted to determine how these interventions have affected CRDMC, as they may have had a positive impact on outcomes and subsequently reduced medical costs.

We have also revealed that the rise in the proportion of CRDMC inpatient health insurance payments among all pediatric inpatients was lower than fee-for-service equivalent medical costs. This implies that, under the current insurance system, admitting CRDMC is becoming disadvantageous to hospital management compared to admitting other children. In remedying this inequity, the payment system should be revised to facilitate more generous payment allocations to CRDMC.

This study highlights problems with the inpatient care of CRDMC, a patient group for which government-led support has developed rapidly in Japan.^49^ As expressed by the Japanese cabinet in 2023,^50^^,^^51^ further support for CRDMC is anticipated to focus on coordinating care between the hospital and the community. Care coordination programs vary with the target population, team and organization structures, payment systems, and program content,^52^^,^^53^ influencing program efficacy.^54^^–^^60^ In planning and evaluating care coordination programs for CRDMC, improvements must focus on overcoming the issues highlighted in this study.

### Limitations

We have several limitations in this study. First, we may have failed to identify some of the CRDMC since there are no validated criteria to identify these children using the DPC database. Although the first requirement in our criteria aimed to identify home-cared CRDMC and the second requirement aimed to identify in-hospital CRDMC, either requirement was underpowered for detecting all the patients. For example, CRDMC in home-based care who were not supervised in the same hospital where they had been hospitalized could not be identified by the first requirement. Furthermore, CRDMC who are receiving daily medical care in settings other than home or hospital (eg, nursing homes) could not be identified using either the first or second requirement. To address this issue, we added the third, fourth, and fifth requirements, which identified CRDMC from different perspectives, in order to supplement the former two requirements. Second, conversely, since some of the codes used to identify CRDMC, such as “unknown” medical care, include a broader range of care than those listed in this study, we may have incorrectly identified some patients who do not require the listed medical care. However, since CRDMC with unknown medical care accounted for only 2.7% of the CRDMC population in this study, the number of incorrectly identified CRDMC is considered small. Third, since we used hospitalization data only from hospitals that provided both inpatient and outpatient data every month with no gaps from April 1, 2013, to March 31, 2022, the number of hospitals included in the study was limited. Thus, the clinical outcomes and medical costs in this study might not fully represent the situation in Japan overall. Nevertheless, the distribution across tiers of pediatric ward classifications in this study was similar to those of a national survey,^61^ except for the lower ratio of hospitals with tier-five wards. We assume that the hospitalization characteristics in hospitals included in this study are fairly likely to represent the whole nation. Fourth, we may have underestimated the length of stay and readmission rate; since we have used a hospital-based database, we could not follow a patient if they were admitted to another hospital. However, we assume that this was a small number of patients since 89.8% of home-cared CRDMC had regular outpatient visits to a specific specialized hospital,^62^ so they were expected to have been hospitalized in that hospital. Fifth, since this study was designed to evaluate the financial impact of CRDMC, rather than to purely characterize the population, the clinical outcomes calculated in this study may have potential limitations. For example, we could not fully address the details of each hospitalization and the heterogeneity within the CRDMC population. These limitations may have affected comparability of the outcomes with non-CRDMC, although we made efforts to reduce these limitations by adjusting the results for covariates, including patients’ underlying diseases.

### Conclusions

From this study, we found that CRDMC had a particularly high 30-day readmission rate. Furthermore, CRDMC accounted for 20.3% of pediatric inpatient medical costs but comprised only 3.7% of total annual unique patients. The proportion of inpatient medical costs increased during the study period, reflecting the increase in patient numbers, whereas costs for injection drugs disproportionately increased relative to patient numbers. Further efforts to improve CRDMC care should be informed by the unique characteristics of this population.

## SUPPLEMENTARY MATERIAL

eMaterial 1. Detailed description of requirements for identifying CRDMC

eTable 1. List of the codes used in each requirement

eTable 2. Characteristics of the included hospitals

eTable 3. Characteristics of CRDMC by the types of care and devices

eTable 4. Clinical outcomes and medical costs of CRDMC by types of care and medical devices

eTable 5. The breakdown of mean inpatient medical costs per day for CRDMC and non-CRDMC hospitalizations

eTable 6. The breakdown of mean inpatient medical costs per hospitalization for CRDMC and non-CRDMC hospitalizations

eTable 7. Estimated proportion of inpatient medical costs, the breakdown of medical costs, and health insurance payments of CRDMC hospitalizations among overall pediatric inpatients by discharge month, based on data from April 2014 to March 2020

eFigure 1. Proportion and estimated increase of the breakdown of inpatient CRDMC medical costs among all pediatric hospitalizations by discharge month. CRDMC, children requiring daily medical care.

eFigure 2. Proportion and estimated increase of inpatient CRDMC medical costs among all pediatric hospitalizations by discharge month, based on data from April 2014 to March 2021 (FY 2014 to FY 2020) and the estimated increase based on data from April 2014 to March 2020 (FY 2014 to FY 2019). CRDMC, children requiring daily medical care; FY, fiscal year.

## References

[1] Cohen E, Kuo DZ, Agrawal R, . Children with medical complexity: an emerging population for clinical and research initiatives. Pediatrics. 2011;127(3):529–538. 10.1542/peds.2010-0910 21339266 PMC3387912

[2] Berry JG, Agrawal RK, Cohen E, Kuo DZ. *The landscape of medical care for children with medical complexity*. Special Report. Overland Park, KS: Children’s Hospital Association; 2013.

[3] Millar K, Rodd C, Rempel G, Cohen E, Sibley KM, Garland A. The clinical definition of children with medical complexity: a modified Delphi study. Pediatrics. 2024;153(6):e2023064556. 10.1542/peds.2023-064556 38804054

[4] Joint Committee of the Japan Children’s Medical and Health Council. Guidelines for physicians treating children in severe condition and children requiring daily medical care (in Japanese). https://www.jpeds.or.jp/uploads/files/jusyouji_iryou_sisin.pdf. Accessed January 26, 2024.

[5] Act on supporting children requiring daily medical care and their families (in Japanese). Japan; 2021. https://elaws.e-gov.go.jp/document?lawid=503AC0000000081. Accessed January 26, 2024.

[6] Technology-dependent children: Hospital v. home care—a technical memorandum (OTA-TM-H-38). U.S. Government Printing Office; 1987.

[7] Spratling R. Defining technology dependence in children and adolescents. West J Nurs Res. 2015;37(5):634–651. 10.1177/0193945914526002 24622153

[8] Brenner M, Alexander D, Quirke MB, . A systematic concept analysis of “technology dependent”: challenging the terminology. Eur J Pediatr. 2021;180(1):1–12. 10.1007/s00431-020-03737-x 32710305 PMC7380164

[9] Yamada H, Ohno K, Shiota M, . Prevalence and clinical characteristics of children with medical complexity in Tottori Prefecture, Japan: a population-based longitudinal study. Brain Dev. 2020;42(10):747–755. 10.1016/j.braindev.2020.06.008 32622762

[10] Yamaoka Y, Tamiya N, Watanabe A, . Hospital-based care utilization of children with medical complexity in Japan. Pediatr Int. 2018;60(7):626–633. 10.1111/ped.13586 29676518

[11] Matsuzawa A, Shiroki Y, Arai J, Hirasawa A. Care coordination for children with medical complexity in Japan: caregivers’ perspectives. Child Care Health Dev. 2020;46(4):436–444. 10.1111/cch.12767 32246855

[12] Sakagami Y, Nakayama N, Konishi K. Reliability and validity of home-visit nursing quality indicators for children with medical complexity in Japan. J Pediatr Nurs. 2022;63:136–142. 10.1016/j.pedn.2021.11.027 34952741

[13] Kosaka M, Murata N, Kaneda Y, . Challenges when going on excursions with children with medical complexity in Japan. Pediatr Int. 2023;65(1):e15403. 10.1111/ped.15403 36318269

[14] Nagura M, Maeda H, Yamamoto M, et al. A new public health method to assess the number of technology-dependent children. Preprints with The Lancet. Preprint posted online June 27, 2023.

[15] Maeda H, Tomomatsu I, Iikura I, . The care burden for technology-dependent children with long-term home ventilation increases along with the improvement of their motor functions. Eur J Pediatr. 2024;183(1):135–147. 10.1007/s00431-023-05249-w 37843613 PMC10858118

[16] Berry JG, Hall DE, Kuo DZ, . Hospital utilization and characteristics of patients experiencing recurrent readmissions within children’s hospitals. JAMA. 2011;305(7):682–690. 10.1001/jama.2011.122 21325184 PMC3118568

[17] Markham JL, Hall M, Goldman JL, . Readmissions following hospitalization for infection in children with or without medical complexity. J Hosp Med. 2021;16(3):134–141. 10.12788/jhm.3505 33617439 PMC7929613

[18] Berry JG, Toomey SL, Zaslavsky AM, . Pediatric readmission prevalence and variability across hospitals. JAMA. 2013;309(4):372–380. 10.1001/jama.2012.188351 23340639 PMC3640861

[19] Leyenaar JK, Schaefer AP, Freyleue SD, . Prevalence of children with medical complexity and associations with health care utilization and in-hospital mortality. JAMA Pediatr. 2022;176(6):e220687. 10.1001/jamapediatrics.2022.0687 35435932 PMC9016603

[20] Gold JM, Hall M, Shah SS, . Long length of hospital stay in children with medical complexity. J Hosp Med. 2016;11(11):750–756. 10.1002/jhm.2633 27378587

[21] Simon TD, Berry J, Feudtner C, . Children with complex chronic conditions in inpatient hospital settings in the United States. Pediatrics. 2010;126(4):647–655. 10.1542/peds.2009-3266 20855394 PMC2962571

[22] Cohen E, Berry JG, Camacho X, Anderson G, Wodchis W, Guttmann A. Patterns and costs of health care use of children with medical complexity. Pediatrics. 2012;130(6):e1463–e1470. 10.1542/peds.2012-0175 23184117 PMC4528341

[23] Berry JG, Hall M, Hall DE, . Inpatient growth and resource use in 28 children’s hospitals: a longitudinal, multi-institutional study. JAMA Pediatr. 2013;167(2):170–177. 10.1001/jamapediatrics.2013.432 23266509 PMC3663043

[24] Srivastava R, Downie J, Hall J, Reynolds G. Costs of children with medical complexity in Australian public hospitals. J Paediatr Child Health. 2016;52(5):566–571. 10.1111/jpc.13152 27329909

[25] Mattiello RMA, Pazin-Filho A, Aragon DC, Cupo P, Carlotti APCP. Impact of children with complex chronic conditions on costs in a tertiary referral hospital. Rev Saude Publica. 2022;56:89. 10.11606/s1518-8787.2022056004656 36259914 PMC9550162

[26] Neff JM, Sharp VL, Muldoon J, Graham J, Myers K. Profile of medical charges for children by health status group and severity level in a Washington State health plan. Health Serv Res. 2004;39(1):73–89. 10.1111/j.1475-6773.2004.00216.x 14965078 PMC1360995

[27] Berry JG, Hall M, Neff J, . Children with medical complexity and Medicaid: Spending and cost savings. Health Aff (Millwood). 2014;33(12):2199–2206. 10.1377/hlthaff.2014.0828 25489039 PMC5164920

[28] Chen LP, Gerber DM, Coller RJ. Admitting what is needed: How the health system and society can reduce hospitalizations for children with medical complexity. J Hosp Med. 2023;18(1):90–94. 10.1002/jhm.12948 35996947 PMC9817383

[29] Hayashida K, Murakami G, Matsuda S, Fushimi K. History and profile of diagnosis procedure combination (DPC): development of a real data collection system for acute inpatient care in Japan. J Epidemiol. 2021;31(1):1–11. 10.2188/jea.JE20200288 33012777 PMC7738645

[30] Yasunaga H, Matsui H, Horiguchi H, Fushimi K, Matsuda S. Clinical epidemiology and health services research using the diagnosis procedure combination database in Japan. Asian Pac J Dis Manag. 2015;7(1–2):19–24. 10.7223/apjdm.7.19

[31] Yasunaga H. Real world data in Japan: Chapter II the diagnosis procedure combination database. Ann Clin Epidemiol. 2019;1(3):76–79. 10.37737/ace.1.3_76

[32] Suzuki Y. New scoring system for patients with severe motor and intellectual disabilities, medical care dependent group. Jpn J Severe Motor Intellect Disabil. 2008;33(3):303.

[33] Kishimoto K, Bun S, Shin JH, . Early impact of school closure and social distancing for COVID-19 on the number of inpatients with childhood non-COVID-19 acute infections in Japan. Eur J Pediatr. Sep 2021;180(9):2871–2878. 10.1007/s00431-021-04043-w 33791861 PMC8012019

[34] Okubo Y, Michihata N, Yoshida K, . Impact of pediatric obesity on acute asthma exacerbation in Japan. Pediatr Allergy Immunol. Dec 2017;28(8):763–767. 10.1111/pai.12801 29044803

[35] Sawakami T. Current status of specific pediatric chronic diseases in Japan: national measures, disease types, treatment availability, copayment assistance, and research. Intractable Rare Dis Res. 2021;10(4):283–287. 10.5582/irdr.2021.01145 34877241 PMC8630460

[36] Moriichi A, Yokoya S. Study on ICD-10 Code Numbering for Specific Pediatric Chronic Diseases (in Japanese). https://www.shouman.jp/research/pdf/r2reports/16_buntan13.pdf. Accessed March 14, 2025.

[37] Buntin MB, Zaslavsky AM. Too much ado about two-part models and transformation? Comparing methods of modeling Medicare expenditures. J Health Econ. 2004;23(3):525–542. 10.1016/j.jhealeco.2003.10.005 15120469

[38] Deb P, Norton EC. Modeling health care expenditures and use. Annu Rev Public Health. 2018;39:489–505. 10.1146/annurev-publhealth-040617-013517 29328879

[39] Markham JL, Richardson T, Hall M, ; Pediatric Research in Inpatient Settings (PRIS) Network. Association of weekend admission and weekend discharge with length of stay and 30-day readmission in children’s hospitals. J Hosp Med. Feb 1 2019;14(2):75–82. 10.12788/jhm.3085 30379138 PMC12820772

[40] Brittan MS, Sills MR, Fox D, . Outpatient follow-up visits and readmission in medically complex children enrolled in Medicaid. J Pediatr. 2015;166(4):998–1005.e1. 10.1016/j.jpeds.2014.12.022 25641248

[41] Howard SW, Zhang Z, Buchanan P, . The effect of a comprehensive care transition model on cost and utilization for medically complex children with cerebral palsy. J Pediatr Health Care. 2017;31(6):634–647. 10.1016/j.pedhc.2017.04.017 28622983

[42] Feinstein JA, Feudtner C, Valuck RJ, Kempe A. The depth, duration, and degree of outpatient pediatric polypharmacy in Colorado fee-for-service Medicaid patients. Pharmacoepidemiol Drug Saf. 2015;24(10):1049–1057. 10.1002/pds.3843 26248529

[43] Feinstein JA, Hall M, Antoon JW, . Chronic medication use in children insured by Medicaid: a multistate retrospective cohort study. Pediatrics. 2019;143(4):e20183397. 10.1542/peds.2018-3397 30914443 PMC6456893

[44] Blaine K, Wright J, Pinkham A, . Medication order errors at hospital admission among children with medical complexity. J Patient Saf. Jan 1 2022;18(1):e156–e162. 10.1097/PTS.0000000000000719 32398538

[45] Feinstein JA, Feudtner C, Kempe A. Adverse drug event-related emergency department visits associated with complex chronic conditions. Pediatrics. 2014;133(6):e1575–e1585. 10.1542/peds.2013-3060 24843054

[46] Feinstein J, Dai D, Zhong W, Freedman J, Feudtner C. Potential drug–drug interactions in infant, child, and adolescent patients in children’s hospitals. Pediatrics. 2015;135(1):e99–e108. 10.1542/peds.2014-2015 25511114

[47] Feinstein JA, Feudtner C, Blackmer AB, . Parent-reported symptoms and medications used among children with severe neurological impairment. JAMA Netw Open. 2020;3(12):e2029082. 10.1001/jamanetworkopen.2020.29082 33306117 PMC7733159

[48] Medical Division, Health Insurance Bureau, Ministry of Health, Labour and Welfare. Overview of the Revision of Medical Fees in Fiscal Year 2016 (in Japanese). https://www.mhlw.go.jp/file/06-Seisakujouhou-12400000-Hokenkyoku/0000115977.pdf. Accessed March 14, 2025.

[49] Ministry of Health, Labour and Welfare. Measures for the support of children requiring daily medical care and their families (in Japanese). https://www.mhlw.go.jp/stf/seisakunitsuite/bunya/hukushi_kaigo/shougaishahukushi/service/index_00004.html. Accessed January 31, 2024.

[50] Children and Families Agency. General principles for child-related measures (in Japanese). https://www.cfa.go.jp/assets/contents/node/basic_page/field_ref_resources/f3e5eca9-5081-4bc9-8d64-e7a61d8903d0/276f4f2c/20231222_policies_kodomo-taikou_21.pdf. Accessed January 14, 2024.

[51] Children and Families Agency. Strategies for future policies on children (in Japanese). https://www.cfa.go.jp/assets/contents/node/basic_page/field_ref_resources/fb115de8-988b-40d4-8f67-b82321a39daf/b6cc7c9e/20231222_resources_kodomo-mirai_02.pdf. Accessed January 14, 2024.

[52] Cohen E, Berry JG, Sanders L, Schor EL, Wise PH. Status complexicus? The emergence of pediatric complex care. Pediatrics. 2018;141(Supplement_3):S202–S211. 10.1542/peds.2017-1284E 29496971

[53] Pordes E, Gordon J, Sanders LM, Cohen E. Models of care delivery for children with medical complexity. Pediatrics. 2018;141(Supplement_3):S212–S223. 10.1542/peds.2017-1284F 29496972

[54] Cohen E, Quartarone S, Orkin J, . Effectiveness of structured care coordination for children with medical complexity: the Complex Care for Kids Ontario (CCKO) randomized clinical trial. JAMA Pediatr. 2023;177(5):461–471. 10.1001/jamapediatrics.2023.0115 36939728 PMC10028546

[55] Simon TD, Whitlock KB, Haaland W, . Effectiveness of a comprehensive case management service for children with medical complexity. Pediatrics. 2017;140(6):e20171641. 10.1542/peds.2017-1641 29192004

[56] Mosquera RA, Avritscher EBC, Samuels CL, . Effect of an enhanced medical home on serious illness and cost of care among high-risk children with chronic illness: a randomized clinical trial. JAMA. 2014;312(24):2640–2648. 10.1001/jama.2014.16419 25536255

[57] Coller RJ, Klitzner TS, Lerner CF, Chung PJ. Predictors of 30-day readmission and association with primary care follow-up plans. J Pediatr. 2013;163(4):1027–1033. 10.1016/j.jpeds.2013.04.013 23706518

[58] Bergman DA, Keller D, Kuo DZ, . Costs and use for children with medical complexity in a care management program. Pediatrics. 2020;145(4):e20192401. 10.1542/peds.2019-2401 32229620

[59] Cohen E, Lacombe-Duncan A, Spalding K, . Integrated complex care coordination for children with medical complexity: a mixed-methods evaluation of tertiary care-community collaboration. BMC Health Serv Res. 2012;12(1):366. 10.1186/1472-6963-12-366 23088792 PMC3529108

[60] Casey PH, Lyle RE, Bird TM, . Effect of hospital-based comprehensive care clinic on health costs for Medicaid-insured medically complex children. Arch Pediatr Adolesc Med. 2011;165(5):392–398. 10.1001/archpediatrics.2011.5 21300650

[61] Medical Division, Health Insurance Bureau, Ministry of Health, Labour and Welfare. Central social insurance medical council general meeting (551st), individual matters (part 1) (in Japanese). https://www.mhlw.go.jp/content/12404000/001135957.pdf. Accessed January 29, 2024.

[62] Mizuho Information & Research Institute, Inc. Survey on children in need of home medical care (in Japanese). https://www.mhlw.go.jp/file/06-Seisakujouhou-12200000-Shakaiengokyokushougaihokenfukushibu/0000130383.pdf. Accessed February 4, 2024.

