# Erratum for “Clinical Outcomes and Medical Costs of Hospitalized Children Requiring Daily Medical Care in Japan”

**DOI:** 10.2188/jea.JE20250709

**Published:** 2026-09-05

**Authors:** Osamu Matsumura Momo, Susumu Kunisawa, Kenji Kishimoto, Kiyohide Fushimi, Yuichi Imanaka

**Affiliations:** 1 Department of Healthcare Economics and Quality Management, School of Public Health, Graduate School of Medicine, Kyoto University, Kyoto, Japan; 2 Department of Health Policy and Informatics, Institute of Science Tokyo Graduate School of Medical and Dental Sciences, Tokyo, Japan; 3 Department of Health Security System, Center for Health Security, Graduate School of Medicine, Kyoto University, Kyoto, Japan

In the original publication of the article,^1^ there were two errors due to oversights during the proofreading process: (1) a missing condition for the extraction of hospitalizations in the “Participants” subsection of the Methods section, and (2) reversed assignment of missing data values between non-Children Requiring Daily Medical Care (CRDMC) and CRDMC hospitalizations in footnote b of Table 3 of the original manuscript. Table 1 shows the correction to the main text.

**Table 1.** Erratum

Section	Before correction	After correction
(1) “Participants” subsection of the Methods section	**Participants** Data from hospitals that provided both inpatient and outpatient data every month between April 1, 2013, and March 31, 2022, was extracted from the DPC database. All extracted data were used for identifying CRDMC. All hospitalizations of children younger than 18 years of age at admission who were discharged between April 1, 2014 (the first day of fiscal year [FY] 2014 in Japan) and March 31, 2021 (the last day of FY 2020 in Japan) were included in the study. The discharge date of each hospitalization was used as the index date. All hospitalizations, including those of the same patients, were analyzed as independent hospitalizations. Hospitalizations with missing claims data were excluded from the study.	**Participants** Data from hospitals that provided both inpatient and outpatient data every month between April 1, 2013, and March 31, 2022, was extracted from the DPC database. **Data for hospitalizations of patients who were admitted before April 1, 2011, was not extracted.** All extracted data were used for identifying CRDMC. All hospitalizations of children younger than 18 years of age at admission who were discharged between April 1, 2014 (the first day of fiscal year [FY] 2014 in Japan) and March 31, 2021 (the last day of FY 2020 in Japan) were included in the study. The discharge date of each hospitalization was used as the index date. All hospitalizations, including those of the same patients, were analyzed as independent hospitalizations. Hospitalizations with missing claims data were excluded from the study.
(2) Table 3, footnote b	Missing data for 30-day non-elective readmissions: 150 in the CRDMC hospitalizations, 24 in the non-CRDMC hospitalizations, 11 in CRDMC receiving a single type of medical care, 13 in CRDMC receiving multiple types of medical care, 23 in home-cared CRDMC, and 3 in in-hospital CRDMC.	Missing data for 30-day non-elective readmissions: 150 in the **non-CRDMC hospitalizations**, 24 in the **CRDMC hospitalizations**, 11 in CRDMC receiving a single type of medical care, 13 in CRDMC receiving multiple types of medical care, 23 in home-cared CRDMC, and 3 in in-hospital CRDMC.

CRDMC, Children Requiring Daily Medical Care.

We sincerely apologize for these errors. The first correction clarifies the Methods section, and the second corrects the reporting of missing data values in Table 3. These were errors in reporting only and did not affect the findings or conclusions of the study.
