# Supplementary material for: Clinical Outcomes and Medical Costs of Hospitalized Children Requiring Daily Medical Care in Japan
Source: J Epidemiol. 2025 Dec 5;35(12):499–509. doi: 10.2188/jea.JE20240457 (PMC12620547; doi:10.2188/jea.JE20240457)

eMaterial 1. Detailed description of requirements for identifying CRDMC

1. Patients with home medical care who are supervised or provided with their medical equipment by the physician at the hospital of hospitalization within 365 days before or after discharge:

(A) patients who were charged supervision fees or additional fees related to home medical care within 365 days before and after the date of discharge from the hospital;

(B) patients who received or were treated with medical equipment which is related to their medical care (including tracheostomy tube, nutrition tube/gastric tube, gastrostomy catheter, enterostomy catheter, infusion kit for central parenteral nutrition, nephrostomy catheter, urostomy catheter, vesicostomy catheter, urethral catheter, equipment for blood dialysis, and equipment for peritoneal dialysis), intravenous hyperalimentation formulas, or dialysis performed during outpatient visits within 365 days before and after the date of discharge from the hospital.

2. Patients who required medical care for more than four consecutive weeks during the hospitalization with no special/specified hospital fees or additional fees for the maternal-fetal intensive care unit, neonatal intensive care unit, growing care unit, intensive care unit, high care unit, or emergency care ward within that period:

(A) patients who received the same medical care (including mechanical ventilation, oxygen therapy, sputum suction, tube feeding, central parenteral nutrition, urethral catheterization, and intestinal cleansing) for a minimum of 28 consecutive days during hospitalization, with no claims for special/specified hospital fees or additional charges for maternal-fetal intensive care unit, neonatal intensive care unit, growing care unit, intensive care unit, high care unit, or emergency care ward during that period;

(B) patients who were treated with medical equipment related to the same medical care (including tracheostomy tube, gastrostomy catheter, enterostomy catheter, nephrostomy catheter, urostomy catheter, vesicostomy catheter, urethral catheter, equipment for blood dialysis, and equipment for peritoneal dialysis) at intervals of at least 28 days during hospitalization, with no claims for special/specified hospital

fees or additional charges for maternal-fetal intensive care unit, neonatal intensive care unit, growing care unit, intensive care unit, high care unit, or emergency care ward during that period.

3. Patients who required certain medical care upon discharge to their home or a nursing home:

(A) patients who were not admitted for same-day surgery and undergone mechanical ventilation, tracheostomy tube replacement, gastrostomy catheter replacement, enterostomy catheter replacement, central parenteral nutrition, nephrostomy catheter replacement, urostomy catheter replacement, vesicostomy catheter replacement, intestinal cleansing, or dialysis or were treated with medical equipment related to these medical procedures (including tracheostomy tube, gastrostomy catheter, enterostomy catheter, infusion kit for central parenteral nutrition, nephrostomy catheter, urostomy catheter, vesicostomy catheter, equipment for blood dialysis, and equipment for peritoneal dialysis) on the day of or a day before discharge;

(B) patients who were not admitted for same-day surgery and had undergone tube feeding or urethral catheterization or were treated with medical equipment related to these medical procedures (including nutrition tube/gastric tube and urethral catheter) on the day of discharge.

4. Patients who had surgery during hospitalization to install or uninstall a medical device used for medical care:

(A) patients who underwent surgical procedures for tracheostomy, gastrostomy, enterostomy, colostomy, nephrostomy, urostomy, or vesicostomy but who had not undergone a closure procedure of those;

(B) patients who did not undergo the aforementioned surgical procedures but who underwent a closure procedure of those during hospitalization.

5. Patients who were charged an extra fee for scoring ten or more points on the Severe Motor and Intellectual Disabilities-Medical Care Dependent Group Scoring System,[32] which suggested that the

patient was receiving at least one of the listed types of medical care:

patients who were charged an extra fee for patients with semi-extreme (scoring between 10 and 24 points on the Severe Motor and Intellectual Disabilities-Medical Care Dependent Group Scoring System) or extremely severe (scoring 25 points or more on the aforementioned system) home medical care, during the relevant hospitalization.

eTable 1. List of the codes used in each requirement

Requirement No		Claim codes (in 9 digits) or drug codes (in 7 digits) used in the requirement
**1.**	**A)**	114005410, 114005510, 114006810, 114009610, 114009710, 114010370, 114017310, 114040710, 114040810, 114041210, 114041310, 114041710, 114045670, 114011110, 114011210, 114050910, 114003710, 114004110, 114004910, 114005010, 114006110, 114006210, 114006310, 114015710, 114041510, 114041610, 114042770, 114043670, 114045470, 114050170, 114053150, 114020910, 114004310, 114005210, 114021210, 114045510, 114004210, 114005110, 114004410, 114008970, 114040910, 114041010, 114041110, 114050310, 114050410, 114050510, 114050610, 114045810, 114045970, 114050710, 140038310, 140038410, 113002510, 114009310, 114009410, 114009510, 190153090, 190153190, 190153290, 190153390, 114003510, 114003610, 114006510, 114006610, 114008250, 114061370, 114017210, 114005810
	**B)**	710010795, 732730000, 710010796, 732740000, 710010797, 732750000, 710010798, 732760000, 710010799, 732770000, 710010800, 732780000, 710010801, 732790000, 710010970, 733830000, 710010971, 733840000, 710010972, 733850000, 710010973, 733860000, 710010974, 733870000, 710010975, 733880000, 710010976, 733890000, 710011086, 710011103, 710011087, 710011104, 710011088, 710011105, 710011089, 710011106, 710011090, 710011107, 710011091, 710011118, 710011158, 710011159, 710010076, 721002000, 710010077, 721003000, 710011078, 721005000, 721004000, 733810000, 721006000, 733820000, 732840000, 738000000, 733260000, 140051210, 140054710, 710010072, 710010091, 710010073, 710010092, 710010074, 710010144, 710010075, 710010145, 710010143, 733220000, 710010146, 733240000, 710010147, 733250000, 710010151, 733300000, 710010152, 733310000, 710010153, 733320000, 710010635, 732800000, 737960000, 732820000, 737980000, 732830000, 737990000, 733330000, 710010069, 710010088, 710010070, 710010089, 710010071, 710010090, 140013810, 140013950, 710010802, 750080000, 710010803, 728370000, 710010804, 728380000, 710010805, 728390000, 710010806, 728400000, 710010897, 710010980, 710010898, 710010981, 710010023, 710010913, 710010914, 710010915, 710010916, 710010917, 721001000, 733740000, 733750000, 733760000, 738260000, 738270000, 738280000, 738290000, 738300000, 738310000, 738320000, 738330000, 140051310, 140007710, 140007910, 140008170, 140033770, 140036710, 140051010, 140051110, 140052570, 140052810, 140052970, 140057810, 140057910, 140058010, 140058110, 140058210, 140058310, 140058410, 140058510, 140058610, 140058770, 140058870, 140058970, 140059070, 140059170, 140059310, 140059410, 140059510, 140060210, 140060310, 140060410, 140060510, 140060610, 140060710, 140060810, 140060910, 140061010, 140062870, 190167970, 190242610, 710010817, 730800000, 710010818, 730810000, 710010828, 710010829, 710010899, 710010982, 710010900, 710010983, 710010901,

		710010984, 710010902, 710010985, 710010903, 710010986, 710010904, 710010987, 710010905, 710010988, 710010906, 710010989, 710010907, 710010990, 710010908, 710010991, 710010928, 710010929, 710010930, 710010931, 710010932, 710010933, 710010934, 710010935, 710010936, 710010937, 720050000, 720060000, 720110000, 140008510, 140008770, 140008810, 140054850, 140054950, 710010654, 732670000, 721018000, 732680000, 737900000, 732690000, 737910000, 732700000, 737920000, 734000000, 734010000, 734020000, 734030000, 3239502, 3239507, 3239503, 3239514, 3239513, 3239512, 3239515, 3239516, 3239508, 3239509, 3239527, 3259530, 3259531, 3259533, 3259534, 3259523, 3259524, 3259525, 3259520, 3259519, 3259516, 3259517, 3259535, 3259536, 3259526, 3259527, 3259515, 3259522, 3259521
**2.**	**A)**	140006050, 140009310, 140023510, 140039550, 140039650, 140063810, 140064750, 140037810, 140005610, 140005810, 140009650, 140023850, 140064150, 140003810, 140023210, 140023350, 140057070, 140006610, 140037610, 140013110, 140013250, 140013350, 140013410, 140013610, 140014010, 140014210, 140014410, 140029950, 140037110, 3239502, 3239507, 3239503, 3239514, 3239513, 3239512, 3239515, 3239516, 3239508, 3239509, 3239527, 3259530, 3259531, 3259533, 3259534, 3259523, 3259524, 3259525, 3259520, 3259519, 3259516, 3259517, 3259535, 3259536, 3259526, 3259527, 3259515, 3259522, 3259521
	**B)**	140051310, 710010076, 721002000, 710010077, 721003000, 710011078, 721005000, 721004000, 733810000, 721006000, 733820000, 732840000, 738000000, 733260000, 710010795, 732730000, 710010796, 732740000, 710010797, 732750000, 710010798, 732760000, 710010799, 732770000, 710010800, 732780000, 710010801, 732790000, 710010970, 733830000, 710010971, 733840000, 710010972, 733850000, 710010973, 733860000, 710010974, 733870000, 710010975, 733880000, 710010976, 733890000, 710011086, 710011103, 710011087, 710011104, 710011088, 710011105, 710011089, 710011106, 710011090, 710011107, 710011091, 710011118, 710011158, 710011159, 710010023, 710010913, 710010914, 710010915, 710010916, 710010917, 721001000, 733740000, 733750000, 733760000, 738260000, 738270000, 738280000, 738290000, 738300000, 738310000, 738320000, 738330000, 140007710, 140007910, 140008170, 140033770, 140036710, 140051010, 140051110, 140052570, 140052810, 140052970, 140057810, 140057910, 140058010, 140058110, 140058210, 140058310, 140058410, 140058510, 140058610, 140058770, 140058870, 140058970, 140059070, 140059170, 140059310, 140059410, 140059510, 140060210, 140060310, 140060410, 140060510, 140060610, 140060710, 140060810, 140060910, 140061010, 140062870, 190167970, 190242610, 710010817, 730800000, 710010818, 730810000, 710010828, 710010829, 710010899, 710010982, 710010900, 710010983, 710010901, 710010984, 710010902, 710010985, 710010903, 710010986, 710010904, 710010987, 710010905, 710010988, 710010906, 710010989, 710010907,

	710010990, 710010908, 710010991, 710010928, 710010929, 710010930, 710010931, 710010932, 710010933, 710010934, 710010935, 710010936, 710010937, 720050000, 720060000, 720110000, 140008510, 140008770, 140008810, 140054850, 140054950, 710010654, 732670000, 721018000, 732680000, 737900000, 732690000, 737910000, 732700000, 737920000, 734000000, 734010000, 734020000, 734030000
**3.**	140006050, 140009310, 140023510, 140039550, 140039650, 140063810, 140064750, 140037810, 710010795, 732730000, 710010796, 732740000, 710010797, 732750000, 710010798, 732760000, 710010799, 732770000, 710010800, 732780000, 710010801, 732790000, 710010970, 733830000, 710010971, 733840000, 710010972, 733850000, 710010973, 733860000, 710010974, 733870000, 710010975, 733880000, 710010976, 733890000, 710011086, 710011103, 710011087, 710011104, 710011088, 710011105, 710011089, 710011106, 710011090, 710011107, 710011091, 710011118, 710011158, 710011159, 140023210, 140023350, 140057070, 140051210, 140054710, 710010072, 710010091, 710010073, 710010092, 710010074, 710010144, 710010075, 710010145, 710010143, 733220000, 710010146, 733240000, 710010147, 733250000, 710010151, 733300000, 710010152, 733310000, 710010153, 733320000, 710010635, 732800000, 737960000, 732820000, 737980000, 732830000, 737990000, 733330000, 710010076, 721002000, 710010077, 721003000, 710011078, 721005000, 721004000, 733810000, 721006000, 733820000, 732840000, 738000000, 733260000, 710010069, 710010088, 710010070, 710010089, 710010071, 710010090, 140013110, 140013250, 140013350, 140013410, 140013610, 140014010, 140014210, 140014410, 140029950, 140037110, 140013810, 140013950, 710010802, 750080000, 710010803, 728370000, 710010804, 728380000, 710010805, 728390000, 710010806, 728400000, 710010897, 710010980, 710010898, 710010981, 710010023, 710010913, 710010914, 710010915, 710010916, 710010917, 721001000, 733740000, 733750000, 733760000, 738260000, 738270000, 738280000, 738290000, 738300000, 738310000, 738320000, 738330000, 140051310, 140006610, 140037610, 140007710, 140007910, 140008170, 140033770, 140036710, 140051010, 140051110, 140052570, 140052810, 140052970, 140057810, 140057910, 140058010, 140058110, 140058210, 140058310, 140058410, 140058510, 140058610, 140058770, 140058870, 140058970, 140059070, 140059170, 140059310, 140059410, 140059510, 140060210, 140060310, 140060410, 140060510, 140060610, 140060710, 140060810, 140060910, 140061010, 140062870, 190167970, 190242610, 710010817, 730800000, 710010818, 730810000, 710010828, 710010829, 710010899, 710010982, 710010900, 710010983, 710010901, 710010984, 710010902, 710010985, 710010903, 710010986, 710010904, 710010987, 710010905, 710010988, 710010906, 710010989, 710010907, 710010990, 710010908, 710010991, 710010928, 710010929, 710010930, 710010931, 710010932, 710010933, 710010934, 710010935, 710010936, 710010937,

	720050000, 720060000, 720110000, 140008510, 140008770, 140008810, 140054850, 140054950, 710010654, 732670000, 721018000, 732680000, 737900000, 732690000, 737910000, 732700000, 737920000, 734000000, 734010000, 734020000, 734030000
**4.**	150106210, 150345310, 150345410, 150108410, 150108550, 150184510, 150185710, 150186110, 150186210, 150186350, 150186450, 150389610, 150402470, 150402570, 150420070, 150420170, 150440570, 150440670, 150440770, 150440870, 150440970, 150441070, 150442610, 150185610, 150420310, 150420410, 150420510, 150171610, 150380510, 150171710, 150378110, 150400910, 150401010, 150409610, 150184310, 150364010, 150185210, 150185310, 150185410, 150185510, 150402110, 150402210, 150201110, 150195610, 150420710, 150195910, 150197810, 150197910, 150403910, 150404010, 150201510
**5.**	190076570, 190076670, 190127510, 190127610, 190136170

eTable 2. Characteristics of the included hospitals

FY	Hospital beds, Median (IQR)	Hospitals with NICU, n (%)	Pediatric ward classification[a], n (%)					Missing (n)
			Tier 1	Tier 2	Tier 3	Tier 4	Tier 5	
**2014**	367 (242 – 604)	57 (24.2%)	7 (3%)	50 (21.2%)	16 (6.8%)	53 (22.5%)	7 (3%)	47
**2015**	363 (240 – 602)	57 (24.2%)	8 (3.4%)	48 (20.3%)	17 (7.2%)	54 (22.9%)	10 (4.2%)	47
**2016**	358 (240 – 602)	56 (23.7%)	9 (3.8%)	52 (22%)	16 (6.8%)	51 (21.6%)	10 (4.2%)	47
**2017**	357 (230 – 600)	57 (23.8%)	8 (3.3%)	55 (23%)	17 (7.1%)	46 (19.2%)	12 (5%)	44
**2018**	313 (199 – 476)	37 (19%)	7 (3.6%)	17 (8.7%)	17 (8.7%)	42 (21.5%)	14 (7.2%)	88
**2019**	396 (246 – 600)	68 (25.6%)	12 (4.5%)	57 (21.4%)	17 (6.4%)	62 (23.3%)	15 (5.6%)	17
**2020**	397 (247 – 600)	70 (25.4%)	15 (5.4%)	53 (19.2%)	14 (5.1%)	74 (26.8%)	17 (6.2%)	7

[a]Pediatric ward of each hospital was classified into tier 1 to 5 based on its fulfillment of the following requirements:

Tier 1: 20 or more pediatricians, one nurse per 7 patients, always more than one nurse per 9 patients, dedicated pediatric ward, average length of stay (LOS) less than 20, 200 or more operations for 6 years old or younger, 800 or more pediatric emergency admissions per year

Tier 2: 9 or more pediatricians, one nurse per 7 patients, multiple nurses in the night shift, dedicated pediatric ward, average LOS less than 20, pediatric emergency room open 24 hours a day/7 day a week

Tier 3: 5 or more pediatricians, one nurse per 7 patients, multiple nurses in the night shift, average LOS less than 20, dedicated pediatric ward

Tier 4: 3 or more pediatricians, one nursing staff per 10 patients, multiple nursing staff in the night shift, dedicated pediatric ward

Tier 5: 1 or more pediatricians, one nursing staff per 15 patients

eTable 3. Characteristics of CRDMC by the types of care and devices

A) Characteristics of CRDMC by the types of care

**Characteristic**[a]	**Respiratory care,** n=56,164	**Tube feeding,** n=51,386	**Urinary care,** n=8,845	**Central parenteral nutrition,** n=6,386	**Defecation care,** n=3,904	**Dialysis,** n=1,787	**Congenital intractable skin disease care,** n=104	**Unknown**[b], n=2,500
Age, Median (IQR), y	3 (1 – 9)	3 (1 – 8)	5 (1 – 11)	6 (2 – 11)	0 (0 – 4)	7 (3 – 13)	7 (3 – 12)	2 (0 – 7)
Sex, Number (%)								
-Male	30,688 (54.6)	26,678 (51.9)	4,535 (51.3)	3,199 (50.1)	2,298 (58.9)	960 (53.7)	81 (77.9)	1,464 (58.6)
-Female	25,476 (45.4)	24,708 (48.1)	4,310 (48.7)	3,187 (49.9)	1,606 (41.1)	827 (46.3)	23 (22.1)	1,036 (41.4)
Weight, Median (IQR), kg	11.2 (7.1 – 18.2)	10.5 (6.6 – 16.9)	15.1 (9.4 – 24.8)	16.3 (10.8 – 27.4)	7.7 (3.4 – 14.2)	18.4 (10.8 – 31.4)	14.4 (12.1 – 21.1)	10.1 (3.2 – 17.0)
(Missing, Number)	(1,270)	(1,234)	(187)	(45)	(30)	(8)	(1)	(38)
Admission, Number (%)								
- Directly after birth	3,527 (6.3)	4,083 (7.9)	243 (2.7)	121 (1.9)	549 (14.1)	37 (2.1)	-[c]	528 1.1)
- Born at 32 weeks of gestation or before	1,275 (2.3)	1,229 (2.4)	24 (0.3)	34 (0.5)	203 (5.2)	-[c]	-[c]	280 (11.2)
- (Missing)	(37)	(36)	(3)	(2)	(5)	(0)	(1)	(3)
- Emergency	25,717 (45.8)	25,434 (49.5)	4,201 (47.5)	2,585 (40.5)	1,448 (37.1)	783 (43.8)	44 (42.3)	1,116 (44.6)
- Scheduled	26,817 (47.7)	21,745 (42.3)	4,384 (49.6)	3,671 (57.5)	1,901 (48.7)	964 (53.9)	57 (54.8)	842 (33.7)
- Other	103 (0.2)	124 (0.2)	17 (0.2)	-[c]	-[c]	-[c]	-[c]	14 (0.6)

Diagnosis, Number (%)								
- Upper Respiratory Infections	903 (1.6)	977 (1.9)	127 (1.4)	30 (0.5)	26 (0.7)	10 (0.6)	-[c]	49 (2.0)
- Lower Respiratory Infections, influenza, or COVID-19	10,278 (18.3)	8,591 (16.7)	766 (8.7)	197 (3.1)	118 (3.0)	65 (3.6)	-[c]	334 (13.4)
- Gastrointestinal infections	965 (1.7)	1,238 (2.4)	172 (1.9)	129 (2.0)	143 (3.7)	39 (2.2)	-[c]	75 (3.0)
- Urinary tract infections	720 (1.3)	1,108 (2.2)	1,101 (12.4)	34 (0.5)	96 (2.5)	29 (1.6)		30 (1.2)
- Skin, soft tissue infections	155 (0.3)	155 (0.3)	54 (0.6)	-[c]	-[c]	-[c]	11 (10.6)	-[c]
- Asthma	1,463 (2.6)	1,091 (2.1)	82 (0.9)	18 (0.3)	23 (0.6)	10 (0.6)	-[c]	78 (3.1)
- Epilepsies and seizures	2,759 (4.9)	3,230 (6.3)	273 (3.1)	56 (0.9)	25 (0.6)	12 (0.7)		165 (6.6)
Diagnosed with any specific pediatric chronic diseases	39,240 (69.9)	31,344 (61.0)	5,407 (61.1)	5,441 (85.2)	2,102 (53.8)	1,342 (75.1)	83 (79.8)	1,401 (56.0)
- Malignant neoplasm	2,602 (4.6)	1,905 (3.7)	645 (7.3)	2,183 (34.2)	70 (1.8)	40 (2.2)		173 (6.9)
- Chronic kidney disease	1,011 (1.8)	1,618 (3.1)	1,336 (15.1)	193 (3.0)	169 (4.3)	1,007 (56.4)	3 (2.9)	33 (1.3)
- Chronic respiratory disease	11,879 (21.2)	8,786 (17.1)	897 (10.1)	394 (6.2)	229 (5.9)	136 (7.6)	7 (6.7)	377 (15.1)
- Chronic heart disease	15,060 (26.8)	7,422 (14.4)	393 (4.4)	365 (5.7)	487 (12.5)	142 (7.9)	9 (8.7)	236 (9.4)
- Endocrine disease	3,353 (6.0)	3,189 (6.2)	513 (5.8)	266 (4.2)	111 (2.8)	273 (15.3)	-[c]	112 (4.5)
- Connective tissue disease	143 (0.3)	127 (0.2)	17 (0.2)	38 (0.6)	23 (0.6)	-[c]	-[c]	-[c]
- Diabetes mellitus	261 (0.5)	208 (0.4)	39 (0.4)	33 (0.5)	24 (0.6)	17 (1.0)	-[c]	-[c]
- Inborn error of metabolism	2,464 (4.4)	2,609 (5.1)	382 (4.3)	117 (1.8)	26 (0.7)	-[c]	-[c]	156 (6.2)

- Hematologic disease	455 (0.8)	318 (0.6)	22 (0.2)	146 (2.3)	21 (0.5)	39 (2.2)	-[c]	-[c]
- Immune disease	822 (1.5)	882 (1.7)	71 (0.8)	504 (7.9)	36 (0.9)	58 (3.2)	-[c]	36 (1.4)
- Neuromuscular disease	7,328 (13.0)	7,309 (14.2)	1,233 (13.9)	235 (3.7)	97 (2.5)	85 (4.8)	-[c]	364 (14.6)
- Chronic digestive disease	763 (1.4)	2,250 (4.4)	738 (8.3)	2,300 (36.0)	1,139 (29.2)	34 (1.9)	-[c]	111 (4.4)
- Syndrome involving chromosomal or genetic alterations	5,386 (9.6)	4,887 (9.5)	466 (5.3)	125 (2.0)	181 (4.6)	24 (1.3)	-[c]	86 (3.4)
- Skin disease	118 (0.2)	88 (0.2)	-[c]	-[c]		-[c]	76 (73.1)	-[c]
- Skeletal dysplasia	384 (0.7)	252 (0.5)	34 (0.4)	-[c]	-[c]	-[c]	-[c]	-[c]
- Vascular disease	282 (0.5)	108 (0.2)	10 (0.1)	16 (0.3)		-[c]	-[c]	-[c]
CRDMC with a single type of medical care, n (%)	25,240 (44.9)	18,765 (36.5)	4,019 (45.4)	2,836 (44.4)	2,275 (58.3)	1,116 (62.5)	93 (89.4)	-[c]
CRDMC with multiple types of medical care, n (%)	30,924 (55.1)	32,621 (63.5)	4,826 (54.6)	3,550 (55.6)	1,629 (41.7)	671 (37.5)	11 (10.6)	-[c]
Home-cared CRDMC, n (%)	49,532 (88.2)	42,217 (82.2)	7,839 (88.6)	4,549 (71.2)	2,594 (66.4)	1,604 (89.8)	104 (100.0)	1,527 (61.1)
In-hospital CRDMC, n (%)	6,465 (11.5)	6,945 (13.5)	626 (7.1)	1,931 (30.2)	335 (8.6)	393 (22.0)	-[c]	-

CRDMC, children requiring daily medical care.
[a] Multiple choices allowed, except for "Unidentifiable."
[b] Identified that the patient requires medical care, although the details of the care were not specified.
[c] Columns with under ten hospitalizations were concealed to protect personal information.

B) Characteristics of CRDMC by medical devices

Characteristic[a]	Tracheostomy, n=17,601	Gastrostomy or enterostomy, n=16,160	Nephrostomy, urostomy or vesicostomy, n=1,705	Colostomy, n= 3,094	Peritoneal dialysis, n=1,635
Age, Median (IQR), y	5 (2 – 10)	5 (2 – 10)	4 (0 – 10)	0 (0 – 4)	6 (3 – 13)
Sex, Number (%)					
-Male	9,599 (54.5)	8,551 (52.9)	997 (58.5)	1,876 (60.6)	868 (53.1)
-Female	8,002 (45.5)	7,609 (47.1)	708 (41.5)	1,218 (39.4)	767 (46.9)
Weight, Median (IQR), kg	13.7 (9.1 – 20.1)	13.6 (9.5 – 19.7)	12.7 (7.5 – 23.1)	7.8 (3.5 – 13.6)	17.2 (10.6 – 29.4)
(Missing, Number)	468	355	36	21	6
Admission, Number (%)					
- Directly_after_birth	459 (2.6)	391 (2.4)	101 (5.9)	401 (13.0)	33 (2.0)
Born at 32 weeks of gestation or before	95 (0.5)	86 (0.5)	17 (1.0)	185 (6.0)	-[c]
(Missing)	(6)	(4)	(1)	(4)	
- Emergency	9,386 (53.3)	8,368 (51.8)	708 (41.5)	1,113 (36.0)	710 (43.4)
- Scheduled	42 (0.2)	21 (0.1)	-[b]	6 (0.2)	-[b]
- Other	7,714 (43.8)	7,380 (45.7)	892 (52.3)	1,574 (50.9)	891 (54.5)
Diagnosis, Number (%)					
- Upper Respiratory Infections	244 (1.4)	272 (1.7)	-[b]	14 (0.5)	10 (0.6)
- Lower Respiratory Infections, influenza, or COVID-19	4,225 (24.0)	3,154 (19.5)	91 (5.3)	83 (2.7)	60 (3.7)
- Gastrointestinal infections	278 (1.6)	397 (2.5)	45 (2.6)	97 (3.1)	37 (2.3)
- Urinary tract infections	355 (2.0)	238 (1.5)	151 (8.9)	87 (2.8)	29 (1.8)

- Skin, soft tissue infections	90 (0.5)	61 (0.4)	-[b]	-[b]	-[b]
- Asthma	428 (2.4)	356 (2.2)	-[b]	19 (0.6)	10 (0.6)
- Epilepsies and seizures	800 (4.5)	1,128 (7.0)	10 (0.6)	17 (0.5)	12 (0.7)
Diagnosed with any specific pediatric chronic diseases	11,744 (66.7)	10,087 (62.4)	1,004 (58.9)	1,783 (57.6)	1,211 (74.1)
- Malignant neoplasm	726 (4.1)	448 (2.8)	86 (5.0)	61 (2.0)	22 (1.3)
- Chronic kidney disease	301 (1.7)	376 (2.3)	350 (20.5)	150 (4.8)	952 (58.2)
- Chronic respiratory disease	4,932 (28.0)	3,368 (20.8)	105 (6.2)	198 (6.4)	129 (7.9)
- Chronic heart disease	1,842 (10.5)	1,385 (8.6)	77 (4.5)	456 (14.7)	111 (6.8)
- Endocrine disease	1,446 (8.2)	1,056 (6.5)	51 (3.0)	99 (3.2)	262 (16.0)
- Connective tissue disease	29 (0.2)	40 (0.2)	-[b]	22 (0.7)	-[b]
- Diabetes mellitus	91 (0.5)	110 (0.7)	-[b]	24 (0.8)	14 (0.9)
- Inborn error of metabolism	1,212 (6.9)	1,063 (6.6)	20 (1.2)	23 (0.7)	-[b]
- Hematologic disease	81 (0.5)	88 (0.5)	-[b]	20 (0.6)	24 (1.5)
- Immune disease	184 (1.0)	162 (1.0)	14 (0.8)	33 (1.1)	47 (2.9)
- Neuromuscular disease	2,910 (16.5)	3,023 (18.7)	52 (3.0)	68 (2.2)	82 (5.0)
- Chronic digestive disease	198 (1.1)	925 (5.7)	374 (21.9)	937 (30.3)	23 (1.4)
- Syndrome involving chromosomal or genetic alterations	1,583 (9.0)	1,280 (7.9)	87 (5.1)	142 (4.6)	21 (1.3)
- Skin disease	25 (0.1)	19 (0.1)	-[b]	-[b]	-[b]
- Skeletal dysplasia	168 (1.0)	99 (0.6)	-[b]	-[b]	-[b]
- Vascular disease	194 (1.1)	17 (0.1)	-[b]	-[b]	-[b]

CRDMC with a single type of medical care, n (%)	3,342 (19.0)	3,816 (23.6)	805 (47.2)	1,692 (54.7)	1,054 (64.5)
CRDMC with multiple types of medical care, n (%)	14,259 (81.0)	12,344 (76.4)	900 (52.8)	1,402 (45.3)	581 (35.5)
Home-cared CRDMC, n (%)	16,196 (92.0)	15,460 (95.7)	1,403 (82.3)	2,386 (77.1)	1,558 (95.3)
In-hospital CRDMC, n (%)	2,696 (15.3)	1,614 (10.0)	198 (11.6)	290 (9.4)	298 (18.2)

CRDMC, children requiring daily medical care.
[a] Multiple choices allowed
[b] Columns with under ten hospitalizations were concealed to protect personal information.

eTable 4. Clinical outcomes and medical costs of CRDMC by types of care and medical devices

A) Clinical outcomes and medical costs of CRDMC by types of care

	Respiratory care, n=56,164	Tube feeding, n=51,386	Urinary care, n=8,845	Central parenteral nutrition, n=6,386	Defecation care, n=3,904	Dialysis, n=1,787	Congenital intractable skin disease care, n=104	Unknown[a], n=2,500
Disposition number (%)[b]								
- Death (In-hospital mortality)	1,304 (2.3)	926 (1.8)	82 (0.9)	360 (5.6)	83 (2.1)	110 (6.2)	-[d]	66 (2.6)
- Discharge to nursing home	257 (0.5)	343 (0.7)	37 (0.4)	-[d]	10 (0.3)	13 (0.7)	-[d]	12 (0.5)
- Transferred to another hospital	2,571 (4.6)	2,461 (4.8)	197 (2.2)	235 (3.7)	182 (4.7)	42 (2.4)	-[d]	248 (9.9)
- Discharge to home	51,972 (92.5)	47,610 (92.7)	8,521 (96.3)	5,777 (90.5)	3,628 (92.9)	1,619 (90.6)	102 (98.1)	2,170 (86.8)
- Other/unidentified	59 (0.1)	45 (0.1)	-[d]	-[d]	-[d]	-[d]	-[d]	-[d]
30-d readmissions, number (%)	16,188 (28.8)	15,043 (29.3)	2,418 (27.3)	3,179 (49.8)	835 (21.4)	485 (27.1)	10 (9.6)	560 (22.4)
30-d non-elective readmissions, number (%)[c]	7,614 (13.6)	7,563 (14.7)	1,337 (15.1)	964 (15.1)	400 (10.2)	236 (13.2)	9 (8.7)	212 (8.5)
Medical cost per hospitalization, mean (SD), JPY	2,311,802 (5,774,426)	2,158,612 (5,647,994)	1,506,036 (4,434,722)	5,666,428 (11,887,387)	3,217,721 (5,697,848)	4,267,421 (11,980,248)	1,082,477 (1,695,697)	2,317,577 (4,898,539)
Medical cost per day, mean (SD), JPY	133,014 (468,688)	100,498 (376,529)	78,052 (114,277)	78,283 (77,790)	83,135 (49,402)	85,441 (64,517)	109,047 (263,583)	134,268 (492,364)
Length of stay, mean (SD), d	27.6 (77.7)	29.8 (81.4)	21.6 (72.3)	66.6 (127.9)	38.3 (77.0)	39.2 (101.1)	15.7 (30.2)	24.5 (39.2)
Total medical cost (JPY)	1.3×10^11	1.1×10^11	1.3×10^10	3.6×10^10	1.3×10^10	7.6×10^9	1.1×10^8	5.8×10^9

CRDMC, children requiring daily medical care; JPY, Japanese yen; SD, standard deviation.

[a] Identified that the patient requires medical care, although the details of the care were not specified.

[b] Missing data for disposition: 1 in respiratory care, 1 in tube feeding, and 1 in CRDMC with type of care unknown.

[c] Missing data for 30-d non-elective readmissions: 18 in respiratory care, 18 in tube feeding, 2 in Urinary care, and 1 in central parenteral nutrition.
[d] Columns with under ten hospitalizations were concealed to protect personal information.

B) Clinical outcomes and medical costs of CRDMC by medical devices

	Tracheostomy, n=17,601	Gastrostomy or enterostomy, n=16,160	Nephrostomy, urostomy or vesicostomy, n=1,705	Colostomy, n= 3,094	Peritoneal dialysis, n=1,635
Disposition number (%)[a]					
- Death (In-hospital mortality)	485 (2.8)	222 (1.4)	26 (1.5)	83 (2.7)	54 (3.3)
- Discharge to nursing home	117 (0.7)	133 (0.8)	-[c]	-[c]	13 (0.8)
- Transferred to another hospital	1,262 (7.2)	909 (5.6)	52 (3.0)	177 (5.7)	29 (1.8)
- Discharge to home	15,707 (89.2)	14,878 (92.1)	1,620 (95.0)	2,825 (91.3)	1,536 (93.9)
- Other/unidentified	29 (0.2)	17 (0.1)	-[c]	-[c]	-[c]
30-d readmissions, number (%)	5,239 (29.8)	4,383 (27.1)	473 (27.7)	707 (22.9)	467 (28.6)
30-d non-elective readmissions, number (%)[b]	2,924 (16.6)	2,574 (15.9)	243 (14.3)	339 (11.0)	230 (14.1)
Medical cost per hospitalization, mean (SD), JPY	2,469,147 (6,699,904)	1,785,625 (4,875,088)	2,683,341 (7,269,920)	3,690,362 (6,153,547)	2,730,613 (7,658,107)
Medical cost per day, mean (SD), JPY	136,272 (561,471)	110,411 (449,363)	75,761 (41,738)	87,129 (52,155)	78,937 (52,464)
Length of stay, mean (SD), d	35.1 (106.6)	25.6 (80.8)	37.6 (111.4)	43.0 (81.4)	31.7 (91.0)
Total medical cost (JPY)	4.3×10^10	2.9×10^10	4.6×10^9	1.1×10^10	4.5×10^9

CRDMC, children requiring daily medical care; JPY, Japanese yen; SD, standard deviation.

[a] Missing data for disposition: 1 in tracheostomy, 1 in gastrostomy or enterostomy.
[b] Missing data for 30-d non-elective readmissions: 8 in tracheostomy, 4 in gastrostomy or enterostomy, and 1 in nephrostomy, urostomy or vesicostomy.
[c] Columns with under ten hospitalizations were concealed to protect personal information.

eTable 5. The breakdown of mean inpatient medical costs per day for CRDMC and non-CRDMC hospitalizations

Breakdown of medical cost per day (JPY), Mean (%)	All hospitalizations N=1,531,456	non-CRDMC n=1,440,043	CRDMC n=91,413	Ratio (95% CI)	Adjusted Ratio[a] (95% CI)
**Laboratory and histopathological examinations**	1,361 (1.9)	1,386 (2)	961 (0.9)	0.693 (0.678–0.709)	0.665 (0.651–0.678)
**Imaging tests**	593 (0.8)	604 (0.9)	422 (0.4)	0.698 (0.674–0.722)	0.542 (0.522–0.562)
**Oral and topical drugs**	803 (1.1)	697 (1)	2,476 (2.2)	3.551 (3.438–3.666)	1.898 (1.850–1.948)
**Injection drugs**	8,659 (11.9)	6,724 (9.6)	39,129 (34.8)	5.819 (5.532–6.118)	1.609 (1.580–1.639)
**Medical procedures**	475 (0.7)	423 (0.6)	1,295 (1.2)	3.063 (2.958–3.172)	2.641 (2.546–2.740)
**Surgeries**	16,360 (22.5)	16,366 (23.3)	16,264 (14.5)	0.994 (0.974–1.014)	0.829 (0.814–0.845)
**First visit fees**	150 (0.2)	158 (0.2)	33 (0)	0.212 (0.201–0.223)	0.345 (0.329–0.362)
**Medical supervision fees**	216 (0.3)	224 (0.3)	94 (0.1)	0.419 (0.404–0.433)	0.522 (0.504–0.540)
**Home medical care related fees**	402 (0.6)	113 (0.2)	4,953 (4.4)	43.533 (40.283–47.091)	30.054 (28.136–32.116)
**Basic Hospital fees**	8,542 (11.7)	8,815 (12.5)	4,253 (3.8)	0.482 (0.475–0.490)	0.619 (0.609–0.629)
**Special/specified hospital fees**	35,115 (48.2)	34,666 (49.3)	42,178 (37.6)	1.217 (1.211–1.222)	1.132 (1.126–1.137)
**Other**	165 (0.2)	161 (0.2)	236 (0.2)	1.467 (1.287–1.665)	1.103 (1.033–1.177)

CI, confidence interval; CRDMC, children requiring daily medical care.

[a]Adjusted by age, sex, admission status (admission directly after birth, on emergency, scheduled, or other), and disease groups of specific pediatric chronic diseases

eTable 6. The breakdown of mean inpatient medical costs per hospitalization for CRDMC and non-CRDMC hospitalizations

Breakdown of medical cost per hospitalization (JPY), Mean (%)	All hospitalizations N=1,531,456	non-CRDMC n=1,440,043	CRDMC n=91,413
**Laboratory and histopathological examinations**	10,679 (1.6)	9,185 (1.7)	34,212 (1.5)
**Imaging tests**	5,399 (0.8)	4,689 (0.8)	16,593 (0.7)
**Oral and topical drugs**	6,565 (1)	4,697 (0.8)	35,990 (1.6)
**Injection drugs**	54,070 (8.3)	39,139 (7.1)	289,292 (13)
**Medical procedures**	12,184 (1.9)	7,109 (1.3)	92,138 (4.1)
**Surgeries**	139,833 (21.4)	121,191 (21.8)	433,508 (19.5)
**First visit fees**	821 (0.1)	840 (0.2)	516 (0)
**Medical supervision fees**	1,440 (0.2)	1,378 (0.2)	2,413 (0.1)
**Home medical care related fees**	2,082.3436 (0.3)	551.9862 (0.1)	26,190.2988 (1.2)
**Basic Hospital fees**	69,480 (10.6)	63,199 (11.4)	168,416 (7.6)
**Special/specified hospital fees**	347,517 (53.1)	299,438 (54)	1,104,907 (49.8)
**Other**	4,252 (0.6)	3,491 (0.6)	16,250 (0.7)

CRDMC, children requiring daily medical care.

eTable 7. Estimated proportion of inpatient medical costs, the breakdown of medical costs, and health insurance payments of CRDMC hospitalizations among overall pediatric inpatients by discharge month, based on data from April 2014 to March 2020

	Estimated proportion on April 2014 (95% CI), %	Estimated proportion on March 2020 (95% CI), %	Estimated increase rate per 12 months (95% CI)	Estimated increase rate from April 2014 to March 2020 (95% CI)
**Inpatient medical costs**	18.0 (17.3-18.7)	21.8 (19.6-24.2)	1.0329 (1.0214-1.0444)	1.2108 (1.1335-1.2934)
**Laboratory and histopathological examinations**	17.0 (15.9-18.2)	20.9 (17.4-25.1)	1.0352 (1.0154-1.0554)	1.2271 (1.0945-1.3758)
**Imaging tests**	16.1 (15.2-16.9)	20.3 (17.6-23.4)	1.0402 (1.0245-1.0561)	1.2624 (1.1539-1.3811)
**Oral and topical drugs**	28.5 (27-30.1)	36.1 (31.3-41.7)	1.0408 (1.0252-1.0568)	1.2673 (1.1584-1.3865)
**Injection drugs**	24.3 (22.5-26.2)	37.2 (30.6-45.2)	1.0744 (1.0530-1.0964)	1.5292 (1.3570-1.7240)
**Medical procedures**	41.0 (39.2-42.8)	48.8 (43.5-54.7)	1.0300 (1.0177-1.0424)	1.1910 (1.1094-1.2785)
**Surgeries**	17.7 (16.2-19.4)	18.8 (14.7-23.9)	1.0099 (0.9843-1.0362)	1.0600 (0.9107-1.2341)
**First visit fees**	3.3 (3.1-3.5)	4.1 (3.5-4.7)	1.0349 (1.0186-1.0513)	1.2247 (1.1154-1.3448)
**Medical supervision fees**	10.7 (9.8-11.6)	9.3 (7.4-11.7)	0.9775 (0.9538-1.0017)	0.8739 (0.7559-1.0102)
**Home medical care related fees**	57.9 (53.2-62.9)	86.2 (70.1-106)	1.0696 (1.0476-1.0922)	1.4889 (1.3164-1.6850)
**Basic Hospital fees**	13.0 (11.9-14.1)	15.8 (12.6-19.8)	1.0340 (1.0094-1.0591)	1.2185 (1.0569-1.4047)
**Special/specified hospital fees**	17.6 (17.1-18.1)	19.7 (18.3-21.3)	1.0198 (1.0117-1.0279)	1.1229 (1.0712-1.1771)
**Other**	20.4 (18.5-22.5)	24.2 (18.5-31.5)	1.0292 (1.0004-1.0589)	1.1858 (1.0025-1.4027)
**Health insurance payments**	18.6 (17.8-19.3)	20.7 (18.6-23.1)	1.0190 (1.0075-1.0305)	1.1175 (1.0453-1.1948)

CI, confidence interval; CRDMC, children requiring daily medical care.

eFigure 1. Proportion and estimated increase of the breakdown of inpatient CRDMC medical costs among all pediatric hospitalizations by discharge month. CRDMC, children requiring daily medical care.

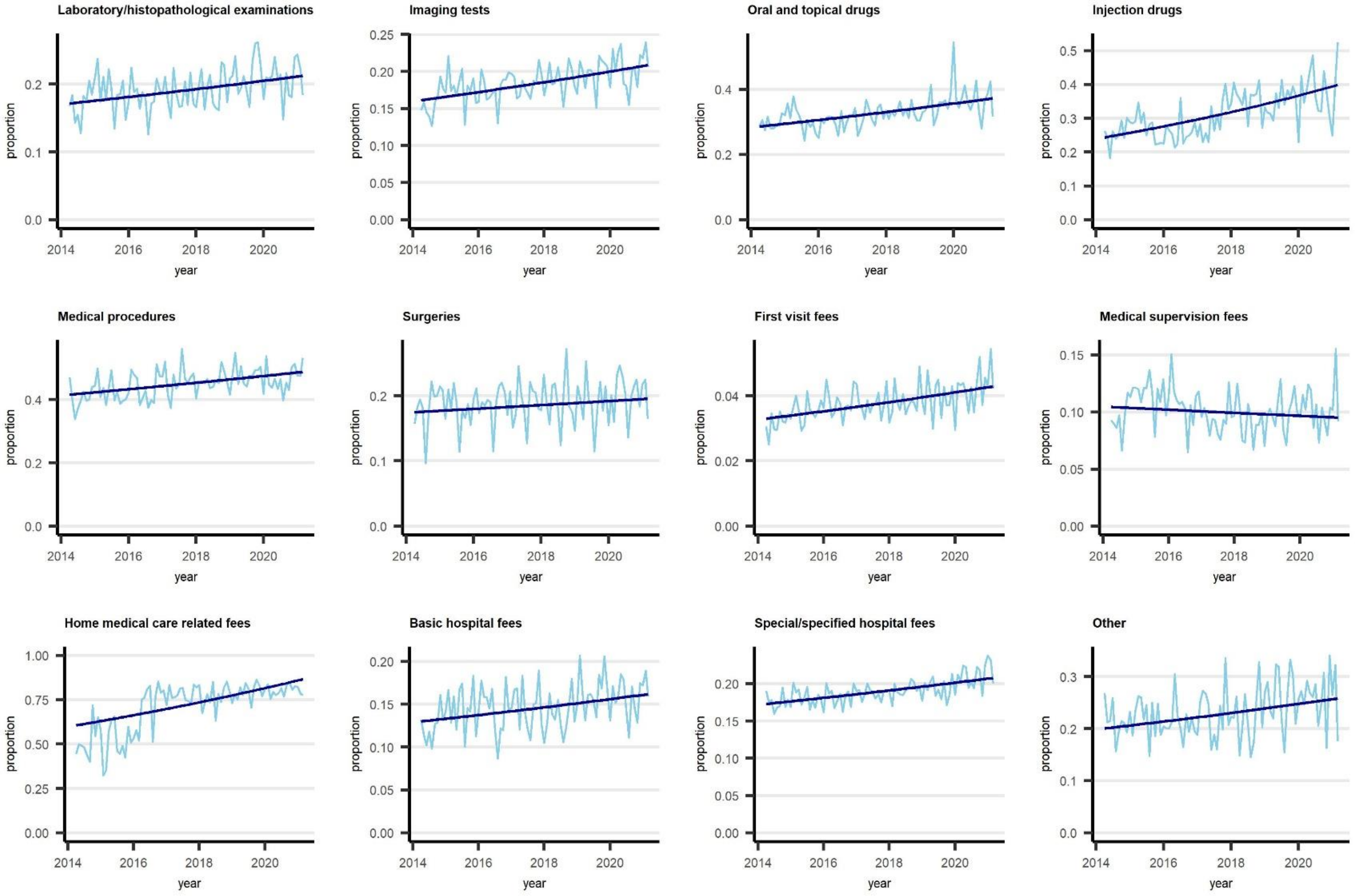

eFigure 2. Proportion and estimated increase of inpatient CRDMC medical costs among all pediatric hospitalizations by discharge month, based on data from April 2014 to March 2021 (FY 2014 to FY 2020) and the estimated increase based on data from April 2014 to March 2020 (FY 2014 to FY 2019). CRDMC, children requiring daily medical care; FY, fiscal year.

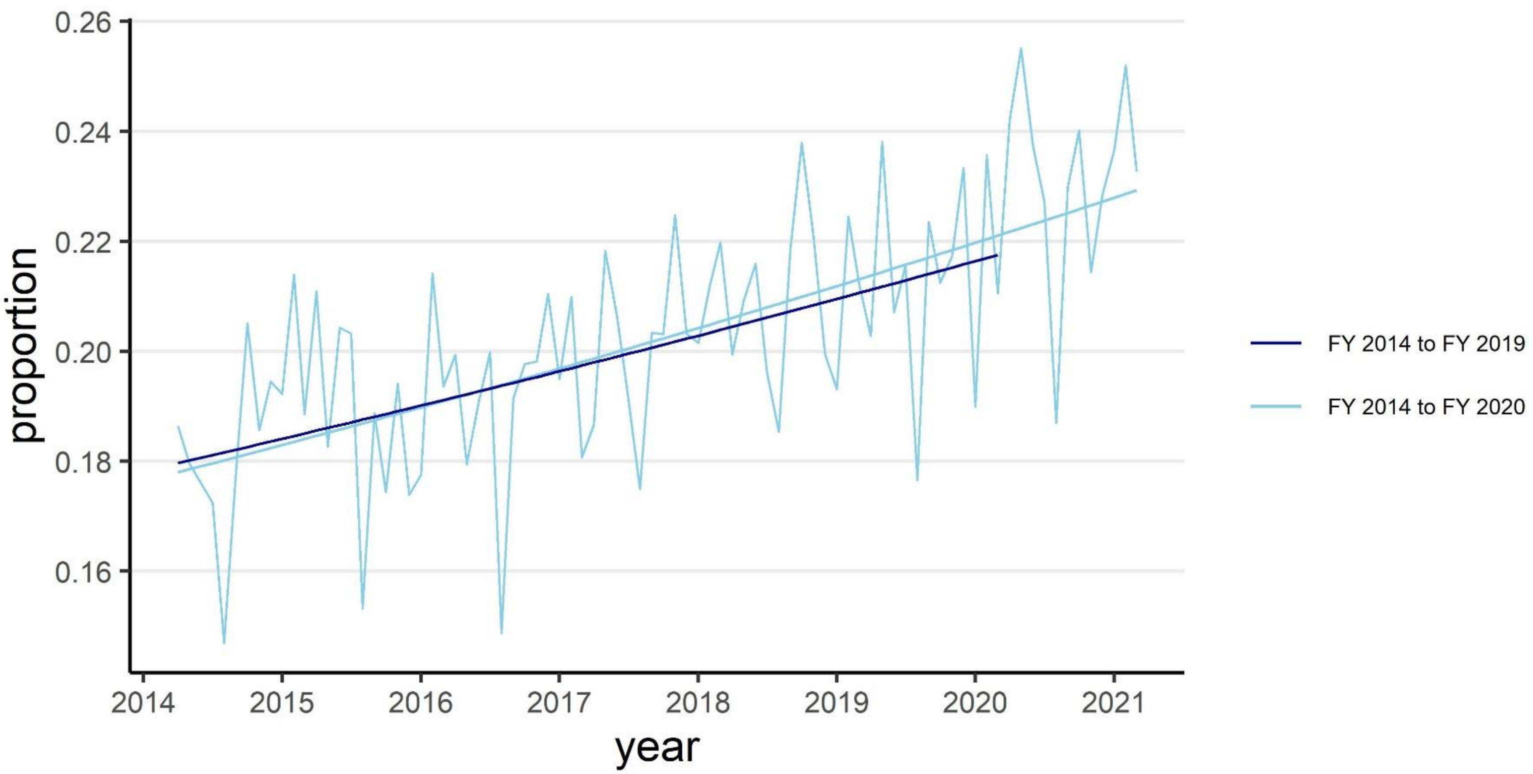

Supplement: Supplementary file 1 [file je-35-499-s001.pdf]
